# Enzyme replacement therapy for the treatment of late onset Pompe disease: A systematic review and network meta-analysis

**DOI:** 10.1186/s13023-025-03981-0

**Published:** 2025-08-21

**Authors:** Mark Corbett, Chinyereugo Umemneku-Chikere, Sarah Nevitt, Nyanar Jasmine Deng, Matthew Walton, Helen Fulbright, Chong Yew Tan, Robin Lachmann, Rachel Churchill, Robert Hodgson

**Affiliations:** 1 Centre for Reviews and Dissemination, University of York, York, UK; 2 Cambridge University Hospitals NHS Foundation, Trust 7, Cambridge, UK; 3 University College London Hospitals NHS Foundation Trust, London, UK

**Keywords:** Glycogen storage disease type II, Enzyme replacement therapy, Network Meta-Analysis, Humans

## Abstract

**Background:**

Late-onset Pompe disease (LOPD) is a rare inherited genetic condition caused by deficiency of acid α-glucosidase (GAA) and accumulation of lysosomal glycogen. LOPD causes progressive muscle dysfunction and damage, leading to significant morbidity and early mortality. Enzyme replacement therapy (ERT) is the primary treatment for Pompe disease.

**Methods:**

A systematic review and network meta-analysis of published evidence on the clinical effectiveness of ERT and best supportive care (BSC) was undertaken to establish the relative effectiveness of ERT compared to BSC (in the absence of ERT). Bibliographic databases were searched to identify randomised controlled trials (RCTs) or any other prospective ERT studies in patients with Pompe disease. Network meta-analyses (NMA) of RCTs were undertaken to estimate indirect treatment effects for forced vital capacity (FVC) % predicted and the 6-minute walk test (6MWD). A narrative synthesis was employed to summarise other studies.

**Results:**

A total of 38 studies were included in the review. They comprised three RCTs, three RCT extension studies, seven registry studies and 25 single-group prospective studies. The results of two RCTs were judged to have a high risk of bias. In the NMA, after approximately one year, ERT-naïve patients showed significant 6MWD improvements vs. placebo: ~25 m with alglucosidase alfa and ~ 54 m with avalglucosidase alfa. No significant differences were found for FVC % predicted or comparisons with cipaglucosidase alfa, although very few ERT-naïve patients taking cipaglucosidase alfa were available for the analyses. Intra-ERT comparisons showed a significant 6MWD advantage for avalglucosidase alfa. However, a sensitivity analysis adjusting for skewed data revealed no significant differences. Long-term ERT effectiveness was assessed in single-group studies, showing initial gains maintained for 1–3 years, followed by gradual 10–15-year declines in 6MWD and FVC % predicted. However, small sample sizes and missing data introduce uncertainty.

**Conclusions:**

Our NMA results showed that ERTs lead to modest improvements in 6MWD after 1 year compared to placebo in ERT-naive populations. However, there is limited evidence supporting meaningful differences in outcomes between ERTs. There is a lack of longer-term follow-up data supporting the effectiveness of ERTs compared to each other and to best supportive care.

**Supplementary Information:**

The online version contains supplementary material available at 10.1186/s13023-025-03981-0.

## Background

Pompe disease, also known as glycogen disease type II, is a rare inherited genetic condition classified as both a glycogen storage and lysosomal storage disorder. It is caused by a deficiency of the enzyme acid alpha-glucosidase (GAA), which is responsible for breaking down glycogen into simpler forms in the body’s cells. This deficiency results in glycogen accumulation within lysosomes, leading to progressive muscle weakness and eventually, muscle damage [1]. 

Pompe disease is classified into two forms: infantile onset Pompe disease (IOPD), with symptoms beginning in the first months of life, and prominent cardiac involvement, and late-(juvenile/adult) onset Pompe disease (LOPD), which can present from early childhood to well into adulthood and primarily affects skeletal muscle [1]. The severity of Pompe disease and age of onset varies widely and is determined, at least in part, by the residual alpha-glucosidase enzyme activity [1]. Most individuals with LOPD undergo a gradual and continuous decline in muscle function, often starting in the proximal muscles, and impacting respiratory function. This deterioration may lead to the need to use mobility aids and ventilatory support. Respiratory failure is the primary cause of premature mortality [2–4]. 

Enzyme replacement therapy (ERT) is the primary treatment for LOPD [5]. ERT involves regular intravenous infusions of recombinant GAA enzyme to help clear glycogen build-up in cells. This can lead to some initial functional improvement and then a slowing of the rate of progression of disease [3, 6]. Eligibility for treatment with ERT typically hinges on a set of criteria, including a confirmed diagnosis, symptomatic presentation of the disease, retention of some level of skeletal and respiratory muscle function, and the absence of another advanced, life-threatening condition. In addition to ERT, patients will also receive supportive treatment, which consists of respiratory support, ambulatory support, physiotherapy, and/or dietary treatment. Patients may also need to consult specialists, including pulmonologists and physical therapists, to manage effectively the various symptoms associated with the condition.

Alglucosidase alfa (ALG) was the first established ERT for the treatment of all types of Pompe disease and has been the standard of care for LOPD since the mid-2000s. More recently, avalglucosidase alfa (AVAL) and cipaglucosidase alfa with miglustat (CM) have become available as alternative ERTs. While several systematic reviews have compared alglucosidase alfa to alternative ERTs [7–12], to our knowledge, no systematic reviews have evaluated the effectiveness of the newly available ERTs and compared them to best supportive care (BSC) without ERT.

Establishing the clinical effectiveness of ERT relative to BSC remains a critical objective, despite ERT having been the mainstay treatment for many years. This is because the drug acquisition costs associated with alglucosidase alfa and other ERTs are exceptionally high, exceeding £250,000 per annum for the average patient. Quantifying the clinical benefits of ERT relative to BSC is essential to determine whether these treatments represent good value for money. This is particularly significant in the UK context, as alglucosidase alfa was commissioned in 2006 by the National Specialised Commissioning Advisory Group as part of the Lysosomal Storage Disorders Service [13–15]. This was prior to the formalisation of National Institute for Health and Care Excellence (NICE) processes for highly specialised technologies and therefore alglucosidase alfa has not been subject to a formal assessment and guidance by NICE [16]. Consequently, more recent appraisals by NICE of avalglucosidase alfa and cipaglucosidase alfa with miglustat have not included BSC as a comparator. The clinical and cost-effectiveness of ERT compared to BSC therefore remains unknown.

This paper presents a systematic review and network meta-analysis (NMA) of all published evidence on the clinical effectiveness of three ERTs used to treat LOPD, considering studies which evaluate their relative effectiveness compared to each other and to supportive care alone, and effectiveness studies without comparators. It reports one component of a National Institute for Health and Care Research (Project number NIHR153779) funded project to evaluate the clinical and cost-effectiveness of ERT for the treatment of LOPD. Other components of the project included an economic analysis of the cost-effectiveness of using ERT to treat LOPD and policy perspectives papers considering the UK approach to evaluating new healthcare technologies. The systematic review was registered on PROSPERO (CRD42024527306), and the full protocol is available online from the NIHR.

## Methods

The review was conducted following the Centre for Reviews and Dissemination guidance on undertaking systematic reviews [17], and results are reported in accordance with the Preferred Reporting Items for Systematic Reviews and Meta-Analyses (PRISMA) statement [18]. 

### Review methods

An Information Specialist (HF) developed an initial search strategy in Ovid MEDLINE with input from the review team. The strategy included terms for Pompe disease with a choice of subject headings and free-text terms. The MEDLINE strategy was adapted as necessary for the other databases and sources searched. No restrictions in terms of study design were applied to any of the searches. Searches were date-limited from 2000 onward and to English language studies. The MEDLINE strategy was peer reviewed by a second Information Specialist with adjustments and corrections made as necessary.

Initial bibliographic searches were undertaken on 12 December 2023 and were updated on 29th May 2024. The following databases were searched: MEDLINE via Ovid; EMBASE via Ovid; KSR Evidence via Ovid; EconLit via Ovid; NHS Economic Evaluations Database (NHS EED) via CRD; Cochrane Database of Systematic Reviews (CDSR) via Wiley; Cochrane Central Register of Controlled Trials (CENTRAL) via Wiley; and the International HTA database via https://database.inahta.org/. Additionally, the following resources were searched for any unpublished, ongoing, or completed studies: ClinicalTrials.gov; European Union Clinical Trials Register; and WHO International Clinical Trials Registry Platform (WHO ICTRP). Searches were conducted on the 12th of December 2023 and later updated in further search on the 29th of May 2024. All references were deduplicated in EndNote 21. The full search strategies for all resources can be found in Additional file 1.

Randomised control trials (RCTs), RCT extension studies (i.e. studies where trial participants have the option of taking the ERT after the randomised phase ends), and other prospectively conducted studies (comparative or single group) that evaluated alglucosidase alfa, avalglucosidase alfa, or cipaglucosidase alfa with miglustat for treating LOPD were eligible. RCTs could be placebo-controlled or head-to-head comparisons. For other comparative studies, BSC had to be evaluated in the absence of a concomitant ERT and had to include one or more of the following: respiratory support (supplemental oxygen), ambulatory support, physiotherapy, and dietary treatment. Studies had to report at least one of the following outcomes: changes in motor function, assessed using the 6-minute walk test distance (6MWD); changes in respiratory function, using forced vital capacity (FVC) % predicted, slow vital capacity, or maximal inspiratory pressure; changes in muscular function, assessed using manual muscle testing, the Gait, Stairs, Gowers’ manoeuvre, and Chair assessment, MRC grading scale, quantitative muscle testing, or the quick motor function test; health-related quality of life (HRQoL); adverse effects; ambulation and ventilator status, including time on ventilator, and mortality. Studies with fewer than 10 patients and those not published in English were excluded.

Titles and abstracts of all identified records were screened independently by two researchers (MC, CUC), as were full publications of potentially relevant studies. Disagreements were resolved through discussion and, where necessary, consultation with a third reviewer.

Risk of bias in RCTs was assessed using the Cochrane Risk of Bias 2.0 tool (RoB 2.0) [19]. Judgements were made based on published papers and associated supplementary files, together with information from European Medicines Agency (EMA) regulatory documents. Data extraction and quality assessment were completed by one reviewer and checked by a second (MC, CUC). Data from studies having multiple publications were consolidated and presented as a single study.

#### Individual participant data

In line with the project protocol, individual participant data (IPD) were sought from eligible RCTs, with sponsors and authors invited to collaborate by contributing anonymised IPD for inclusion in the IPD meta-analysis. Authors or sponsors of the eligible RCTs were contacted either directly or via data-sharing platforms Vivli, Inc depending on the data-sharing process of the sponsor.

#### Methods of synthesis and statistical analysis

A NMA was used to estimate the indirect treatment effect of treatments without head-to-head comparison. The NMA evaluated the relative treatment effectiveness of alglucosidase alfa, avalglucosidase alfa, cipaglucosidase alfa with miglustat, and placebo (BSC) in terms of change from baseline in 6MWD in metres and FVC (% predicted) at varying time points across the included RCTs. For all other outcomes, there were insufficient data to perform a full NMA. The primary analysis focused on outcomes at 49/52 weeks (or the closest available time point) and was conducted in the ERT-naïve population using a random effect estimator. Synthesis of outcomes in an ERT-experienced population was not possible as only the PROPEL trial recruited patients in this population. Aligning time points as closely as possible, separate NMAs were also conducted using results at 12/13 weeks, 24/26 weeks, 37/38 weeks, and the last follow up time for each trial (week 78 in the LOTS trial, week 49 in the COMET trial and week 52 in the PROPEL trial).

A potential outlier in the week 49 COMET trial 6MWD data was identified which appears to have skewed the mean difference between AVAL and ALG at week 49 in favour of AVAL (Additional file 2, Table 3). The range of values of change in 6MWD for the alglucosidase alfa arm (-394.0 m to 193.0 m) indicates that at least one patient experienced a substantial decline in 6MWD and the extreme value(s) seems to have skewed the mean 6MWD in the ALG group at week 49. This skew was evident from the substantial deviation between mean and median values reported for the alglucosidase alfa arm (-1.7 m vs. 16.0 m) [20]. To adjust for this, a sensitivity analysis was conducted which carried forward the COMET week 37 6MWD alglucosidase alfa result, to week 49. This approach was used because the week 37 mean 6MWD result (15.4 m) was very similar to the week 49 median (16 m).

**Fig. 1:**
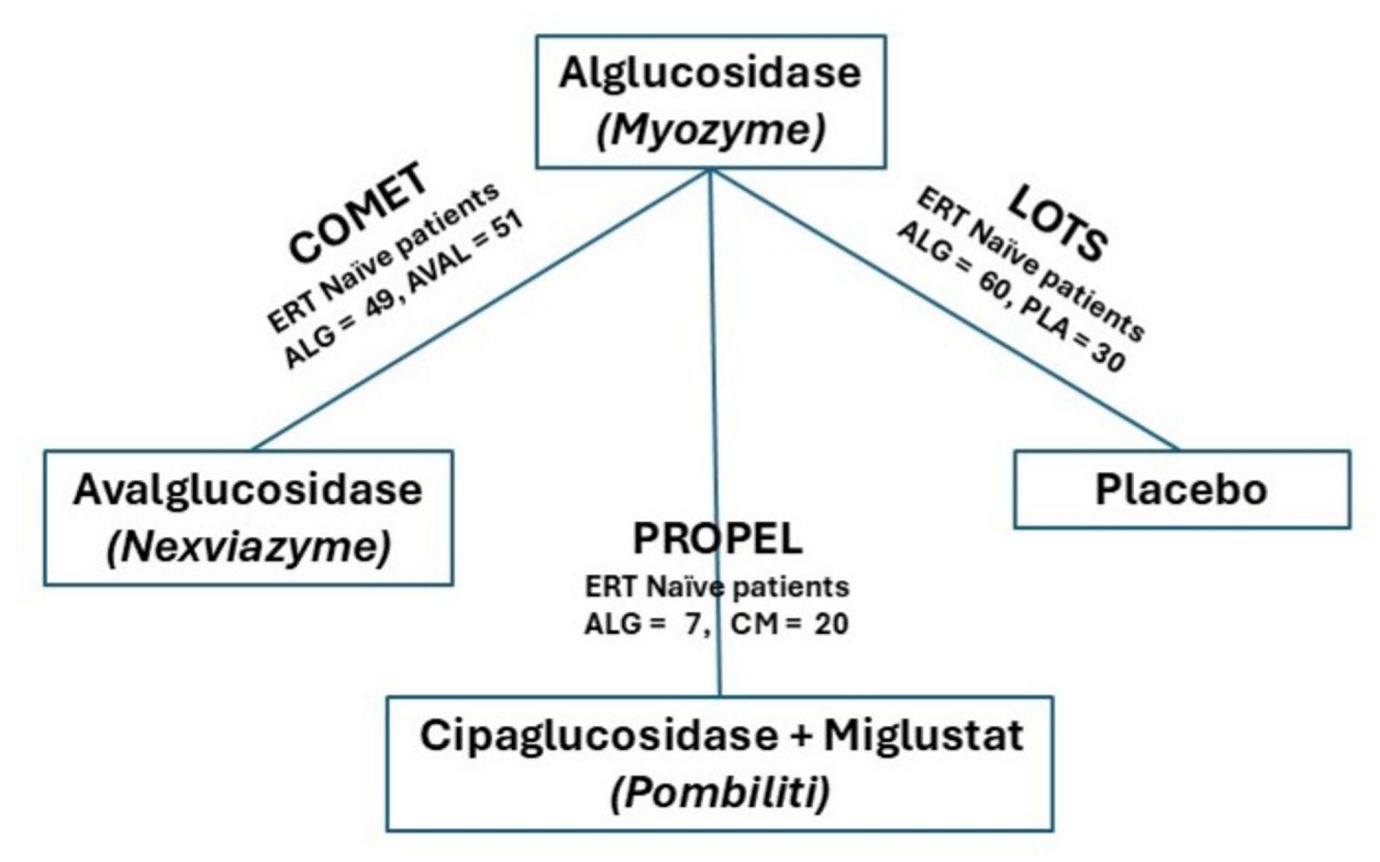
Indirect comparison network

The identified RCT evidence allowed an indirect comparison of all three ERT treatments using alglucosidase alfa as a common comparator, see Fig. 1 for a depiction of the network diagram. Non-RCT evidence was not included in the statistical synthesis due to the high levels of uncertainty associated with incorporating such evidence into an NMA.

The NMA was performed using a Bayesian Markov Chain Monte Carlo (MCMC) approach, implemented in R (version 4.2.3) using the rjags package [21, 22]. The code was based on the NICE Decision Support Unit’s Technical Support Document 2 (DSU TSD 2) [23]. A burn-in of 5,000 iterations was used, followed by three chains run for 500,000 iterations with a thinning interval of 20. Convergence was assessed visually using trace plots and inspection of the Brook-Gelman-Rubin diagnostic value. The estimated relative treatment effects were reported as means with 95% credible intervals (CrI) from their posterior distributions. The R code is provided in the Additional file 2.

Due to the limited number of studies in the NMA, implementing a hierarchical model that incorporates treatment classes—allowing for the estimation of both individual treatment effects and overall class means—was not feasible. Consequently, we assessed the effectiveness of all ERTs combined as a class versus placebo, as well as the effectiveness of individual ERTs compared to each other and to placebo. Both fixed- and random-effects models were evaluated, with between-trial heterogeneity assessed using the between-study standard deviation. However, inconsistency could not be examined, as the network lacked closed loops.

Studies which could not be synthesised quantitatively in an NMA were summarised narratively.

#### Stakeholder involvement

Throughout this project, relevant stakeholder perspectives were properly considered. During protocol development, comments and feedback from two content experts were incorporated. Additionally, two engagement events were held with patient, clinical, and third-sector representatives, including representatives from Pompe UK. This was to facilitate a better understanding, interpretation, and contextualization of the findings of the review.

## Results

### Search results

**Fig. 2:**
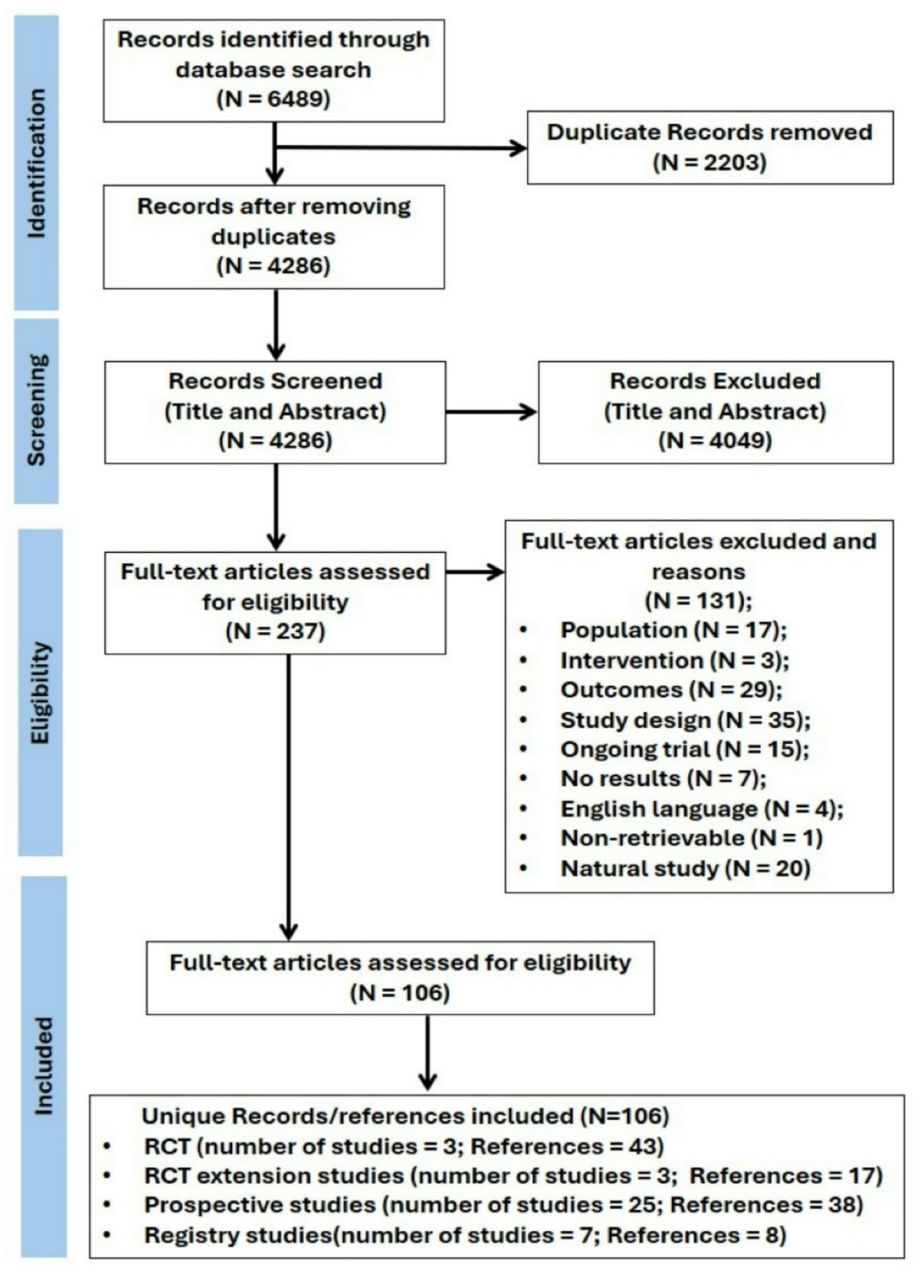
PRISMA flow chart

After screening 4286 titles and abstracts, 237 full-texts were retrieved and screened. Thirty-eight studies (encompassing 106 unique records) were included in the review: three RCTs (LOTS [3], PROPEL [24] and COMET [25]), three RCT extension studies [26–28], seven Pompe disease registry studies [29–35] and 25 single-group prospective studies [2, 6, 36–58]. The process of identifying and selecting records is presented in the PRISMA flowchart (Fig. 2).

### Study details and baseline characteristics

The LOTS RCT evaluated the safety and efficacy of alglucosidase alfa compared to placebo (plus BSC). The COMET and PROPEL RCTs assessed the safety and efficacy of avalglucosidase alfa and cipaglucosidase alfa plus miglustat, respectively, compared to alglucosidase alfa. Mean ages across the three trials ranged from 44 to 48 years (Table 2, Additional file 2). The proportion of participants using a walking aid at baseline was 43% in LOTS and 23% in PROPEL (data were not reported for COMET). LOTS and COMET recruited only ERT-naïve patients, whereas PROPEL recruited mostly ERT-experienced patients.

Of the seven registry studies, there were three studies from Sanofi’s international Pompe Registry cohort [29–31], with sample sizes ranging from 396 to 1390 patients, three studies from the French Pompe disease registry [32–34] (range 29 to 177 patients) and one Spanish Pompe registry study (*N* = 113) [35], (Table 1, Additional file 3). The largest study was reported only as a conference abstract [31]. The mean ages when starting ERT ranged between 45 and 56 years, except for the Spanish registry study which was of a younger cohort (mean 29 years). Follow-up durations ranged from one to 10 years.

All other prospective studies (*n* = 25) included in the review were single-group studies: none compared an ERT with a specific type of best supportive care, although some studies did compare ERT patients with those not taking an ERT. Sample sizes ranged from 11 to 209 patients (Table 3, Additional file 3). Eleven studies included 30 or fewer patients and 6 studies included 100 or more patients; the number of patients included in individual analyses was often notably smaller than the number recruited e.g. some patients were not physically able to undertake an assessment of 6-minute walk distance. Two studies were reported only as conference abstracts [41, 47]. All studies were of alglucosidase alfa, except for the NEO1/NEO-EXT study of avalglucosidase alfa [57], and the ATB200-02 study of cipaglucosidase alfa plus miglustat [58]. Most studies were conducted in Italy, Germany or the Netherlands. Five of the 25 studies [39, 40, 45, 55, 59] reported results for a child cohort or subgroup (mean age ranged between 6 and 12 years), with the remaining studies being of adults (mean age range between 43 and 53 years). In one small study [40] all patients were ERT-experienced at study entry, with patients typically having been on an ERT for around nine years. There was substantial variation across studies in the proportion of patients requiring wheelchair or respiratory support at baseline. Follow up durations ranged from 6 months to 15 years, with most patients being followed up for between 2 and 5 years.

### IPD from eligible trials

IPD was sought from the three identified RCTs: PROPEL, LOTS and COMET. However, no IPD was provided by any of the sponsors. Amicus Therapeutics was contacted regarding sharing data from the PROPEL trial. In email correspondence, Amicus Therapeutics representatives indicated they were working on a process and platform to make these data available but failed to respond to further email contacts. Access to data from the LOTS and COMET trials was sought from the sponsor Sanofi via the data-sharing platform Vivli. Sanofi declined the request stating that they considered the proposed research not to be in the interest of patients or the patient community.

In the absence of IPD from the sponsors of the RCTs, we digitised the mean difference plots of the outcomes (FVC % predicted and 6MWD) using the PlotDigitzer website (https://plotdigitizer.com/) to obtain estimates of the mean differences at 12/13 weeks, 24/25 weeks, 36/38 weeks, and 49/52 weeks.

### Risk of bias assessment

Risk of bias assessment results are presented in Additional file 2, Table 1. The COMET trial results were judged to have a low overall risk of bias but the LOTS and PROPEL trial results were judged to be at high risk of bias. Both LOTS and PROPEL had high risk judgements for the ‘bias in the selection of the reported result’ domain, since both trials failed to report results for all pre-specified analyses, as noted in the respective EMA reports [60]. ^,^ [61]

Another study quality issue identified was the reporting of results using only means in the published reports. Results data from regulatory documents showed skewing of the 6MWD data by outliers, with the means and medians differing substantially. The reporting of only means in the presence of outliers in a sample is not an accurate representation of the efficacy data. For example, the FDA reported that in COMET the mean change in 6MWD from baseline to week 49 for the alglucosidase arm was − 1.7 m, whereas the median was 16.0 m; [20] the EMA reported that in LOTS the mean change in 6MWD from baseline to week 78 for the alglucosidase arm was 26.1 m, whereas the median was 15.0 m [62]. 

### NMAs of RCT evidence

Results from the primary analysis are reported on Table 1 and results of the sensitivity analyses and additional analyses are presented in Additional file 2.

In the primary analysis of FVC % predicted at 49/52 weeks, the results indicate that all three ERTs exhibit numerical superiority over placebo (which includes BSC). However, the estimated mean differences did not reach statistical significance for any of the ERTs (Table 2). In contrast, for 6MWD there were statistically significant improvements compared to placebo for both alglucosidase alfa (by around 25 m) and avalglucosidase alfa (by around 54 m). Cipaglucosidase alfa with miglustat, while numerically superior to placebo, did not show statistically significant differences. Credible intervals for this comparison were wide, reflecting the small number of ERT-naïve patients in the PROPEL trial.

Analysis of additional time points revealed a consistent pattern (Additional File 2 – Tables 8 and 9), with no statistically significant differences between any ERT and placebo for FVC % predicted at any time point. For each ERT versus placebo for 6MWD, statistically significant differences were observed favouring alglucosidase alfa beginning at week 12/13 and persisting through later time points. Similarly, statistically significant differences for avalglucosidase alfa were estimated from week 24/26, with sustained effects observed at subsequent time points. Cipaglucosidase alfa with miglustat showed numerical superiority across all remaining time points, but differences did not reach statistical significance.

Intra-ERT comparisons showed a numerical difference between avalglucosidase alfa and alglucosidase alfa at 49/52 weeks for both FVC% predicted and a meaningful numerical difference in 6MWD. Sensitivity analyses conducted at other time points, including an analysis at 49/52 weeks using imputed values for the COMET trial, similarly did not demonstrate statistically significant differences for either outcome. Results of the sensitivity analysis (Additional File 2, Table 7) also show a smaller numerical difference between avalglucosidase alfa and alglucosidase alfa; mean difference 12.43 m, (95%CrI: -13.17 to 38.07) vs. 28.87 m (95% CrI: 1.74 vs. 55.66). Cipaglucosidase alfa with miglustat exhibited numerical inferiority compared to avalglucosidase alfa for both outcomes across all time points and sensitivity analyses (Additional File 2, Tables 6, 7, 8 and 9); however, the differences were not statistically significant, and credible intervals were wide. Compared to alglucosidase alfa, cipaglucosidase alfa with miglustat demonstrated numerical inferiority for both outcomes in the primary and sensitivity analysis, but the differences were small and credible intervals were wide. This pattern of numerical inferiority was consistent across all time points (except week 24/26) for 6MWD. Differences for FVC% predicted were, however, inconsistent, with weeks 12/13 and 24/26 favouring alglucosidase alfa, and the week 37/38 analysis favouring cipaglucosidase alfa with miglustat. At all-time points and for both outcomes, differences between alglucosidase alfa and cipaglucosidase alfa with miglustat were not statistically significant.

**Table 1:** Relative treatment effects of FVC and 6MWD (random effect NMA), primary analysis (49/52 weeks of follow-up for all RCTs)

Relative treatment effects measured as mean differences (95% credible interval)				
Outcome: Force vital capacity % predicted. Minimum clinically important diference range: 2.5–4.8%^*^				
	PBO	ALG	AVAL	CM
**PBO**		3.58 (-2.95, 10.13)	6.01 (-3.40, 15.33)	3.11 (-6.79, 13.00)
**ALG**	-3.58 (-10.13, 2.95)		2.43 (-4.22, 9.07)	-0.48 (-7.97, 6.98)
**AVAL**	-6.01 (-15.33, 3.40)	-2.43 (-9.07, 4.22)		-2.90 (-12.91, 7.07)
**CM**	-3.11 (-13.00, 6.79)	0.48 (-6.98, 7.97)	2.90 (-7.07, 12.91)	
**Outcome: Six-minute walking distance in metres. Minimum clinically important diference range: 24 m to 57 m** ^*^				
	PBO	ALG	AVAL	CM
**PBO**		24.68 (3.97, 45.65)	53.55 (19.66, 87.31)	19.29 (-17.43, 56.09)
**ALG**	-24.68 (-45.65, -3.97)		28.87 (1.74, 55.66)	-5.39 (-35.72, 25.58)
**AVAL**	-53.55 (-87.31, -19.66)	-28.87 (-55.66, -1.74)		-34.26 (-74.80, 6.96)
**CM**	-19.29 (-56.09, 17.43)	5.39 (-25.58, 35.72)	34.26 (-6.96, 74.80)	

A negative symbol indicates that the treatment in the top row is less effective than the treatment in the first column. ALG, Alglucosidase; AVAL, Avalglucosidase; CM, Cipaglucosidase + Miglustat; PBO, placebo; FVC, forced vital capacity, 6MWD six-minute walking distance; ^*^Sources for minimum clinically important diference ranges are reported in the Discussion

The combined treatment effectiveness of all ERTs demonstrated significant numerical superiority over placebo for 6MWD at all time points (Additional File 2, Table 16). For FVC% predicted, ERTs also showed numerical superiority; however, the difference was not statistically significant using the random-effects model.

Evidence in the ERT-experienced population was limited to a subgroup from the PROPEL trial which included participants who had received ERT for at least 2 years. Results from the PROPEL trial favoured cipaglucosidase alfa with miglustat with statistically significant differences in both 6MWD and FVC% predicted reported, mean difference: 16.8 m (95% CrI: 0.2 to 33.3) and 3.5 (95% CrI: 1.0 to 6.0) respectively. However, the reported results of this trial were judged to be at high risk of bias.

### RCT extension studies

Each of the three RCTs had open-label extension studies. PROPEL was extended by 52 weeks [26], LOTS by 26 weeks (and by 52 weeks for a subset of U.S. patients) [27], and COMET by 48 weeks (reported in a published paper [28]) and 96 weeks (reported in two conference abstracts [63, 64]). Given that patients may potentially receive ERTs for many years, these extension studies are relatively short in terms of providing evidence of the long-term effects of ERTs. Results are reported in Table 14, and 15 of Additional file 2.

In PROPEL, the ERT-experienced group which continued taking cipaglucosidase alfa with miglustat had small increases in % predicted 6MWD and % predicted FVC from week 52 to week 78, followed by small declines by week 104; however, no details were reported on how missing data were handled in the analyses (11 patients discontinued treatment). The LOTS cohort also showed a small decline in 6MWD and FVC % predicted from week 78 to week 104 for the group which continued taking alglucosidase alfa (data were missing for only one patient). In the COMET extension study FVC % predicted remained relatively stable, but 6MWD and hand-held dynamometry had decreased notably by week 97. In mitigation, the authors stated that some patients missed infusions due to the COVID-19 pandemic. A further difficulty when interpreting the COMET extension results is that data were missing at week 97 for 9 (6MWD), and 8 (FVC % predicted), of the 51 patients who continued taking avalglucosidase alfa and the analyses assumed that data were missing at random. This assumption does not appear reasonable for those patients who discontinued due to adverse events.

### Registry studies

Semplicini et al. [32] followed 158 patients for a median of around five years, finding a 1.4% annual increase in % predicted 6MWD up to 2.2 years, followed by a 2.3% decline (Table 2, Additional file 3). For muscle function outcomes, the Motor Function Measurement D2 sub-score showed a progressive 1.0% decline per year, and the D3 sub-score had a slower progressive decline (0.2% per year).

Tard et al. reported on the effect of switching from alglucosidase alfa to avalglucosidase alfa in 29 patients, reporting stabilisation of 6MWD results after one year of avalglucosidase alfa (when compared to pre-switch one year data, which showed declines) [34]. The reporting of 6MWD results data in Lefeuvre et al.’s study was somewhat unclear`, although the treated population experienced a decline in 6MWD. Martinez–Marin et al.’s Spanish registry study reported yearly 6MWD declines of between 5 and 9 m in subgroups treated for < 5 years, 5–10 years and > 10 years [35]. 

All studies reported FVC % predicted, mostly as a long-term outcome. Annual declines in %FVC after up to five years of ERT ranged between 0.17% and 0.9%. Declines at time points between 5 and 13 years were similar across two studies ranging between 1.0 and 1.2% [29, 35]. Tard et al. found no statistically significant difference in %FVC in patients who switched ERT [34]. Three studies reported mortality, with mean or median ages at death ranging between 60 and 66 years [29, 32, 33]. 

### Other prospective studies

#### 6MWD results

The reporting of the 18 studies with 6MWD results varied (Table 5, Additional file 3). Only three studies reported results as medians [39, 48, 53] and only seven reported results as changes from baseline as absolute values; [2, 36, 40, 47, 48, 51, 58] five studies reported results as changes in % predicted 6MWD [41, 46, 55, 57, 58]. The remaining studies either reported results only graphically [49], or reported baseline and end of follow up data but with the difference represented only as a p-value [37–39, 52, 53]. 

Of the studies reporting changes from baseline, two very small studies reported improvements at 6 months of 37 m [2] and 47 m; [47] the result was statistically significant for the former, though level of statistical significance was not reported for the latter. One study reported a statistically significant improvement at up to one year of around 44 m, although this result was also based on a small cohort (*n* = 20) [36]. For later time points, a non-statistically significant improvement of 16 m at > 3 years [36], and a statistically significant increase of 41 m at 5 years [48] were reported. Ravaglia et al. followed a small cohort up to 15 years; although most of the data were only reported graphically, the study’s results showed significant improvement of around 55 m at one year, a return to baseline at around three years, and continued decline up to 15 years [50]. 

The studies reporting % predicted 6MWD results were generally limited by very small samples sizes or by being available only as an abstract. However, Harlaar et al., including 30 patients from the LOTS trial cohort reported initial improvements for around two years, followed by gradual decline up to 10 years [46]. Thirteen patients had some wheelchair dependency at the end of follow-up compared to 7 patients at the start. The remaining 6MWD studies reported statistically significant improvements up to two years and of the two studies which also reported results at three years, one found a statistically significant improvement [39] while the other did not [52]. 

#### Other outcomes

The 16 studies which reported FVC % predicted results were broadly consistent across their results. These indicated little change after up to one or two years of ERT, thereafter followed by slow declines over up to 10 years of follow up (Table 5, Additional file 3). The most common muscle function or strength outcomes reported were the Medical Research Council (MRC) scale (8 studies), handheld dynamometry (HHD, 4 studies) and the quick motor function test (QMFT, 3 studies). The studies reporting MRC scores had heterogeneous results with two studies (both *n* > 50) showing small but statistically significant increases during the first 2–3 years of ERT [36, 42] whereas other studies reporting results for up to three years did not find statistically significant improvements [52, 53]. No statistically significant improvements were seen in any of the studies reporting MRC scores at later time points. Two quite large studies which reported HHD outcomes found statistically significant improvements after up to two years of ERT [42, 48], with one also reporting a plateauing of effect at around three years [48]. These two studies also reported QMFT, which did not improve significantly in either study at up to five years of follow up.

One study analysed the effect of ERT on mortality by comparing ERT patients with patients not taking ERT [44]. It found that the use of ERT was positively and statistically significantly associated with survival. Van der Meijden et al. [54] also compared ERT users with non-ERT users in their large survey study, finding that ERT significantly reduced the risk for wheelchair use, but not the risk of needing respiratory support. Most of the six studies reporting ERT infusion-associated reactions found the occurrence rate to be around 25% (Table 5, Additional file 3).

## Discussion

This review included 38 studies evaluating the effectiveness of ERT for treating Pompe disease. Most of the evidence was derived from single-group studies, including RCT extension studies, prospective cohorts, and registry studies, with a predominant focus on alglucosidase alfa. Comparative evidence was limited to three RCTs which collectively assessed the effectiveness of alglucosidase alfa, avalglucosidase alfa, cipaglucosidase alfa with miglustat and placebo (with BSC).

For the outcomes 6MWD and FVC % predicted, the NMA in ERT-naïve populations demonstrated the effectiveness of alglucosidase alfa and avalglucosidase alfa compared to placebo (BSC) at around one year. However, evidence of the superiority of cipaglucosidase alfa with miglustat remains limited because the PROPEL trial enrolled very few ERT-naive patients. Consequently, the NMA results represent only a subset of the PROPEL participants. However, results from ERT-experienced patients from PROPEL support the effectiveness of cipaglucosidase alfa with miglustat in patients who had previously taken alglucosidase alfa for several years. Our NMAs also suggested that avalglucosidase alfa may be superior to alglucosidase alfa, with statistically significant improvements in 6MWD observed at around 52 weeks and numerical (but not statistically significant) improvements in FVC% predicted. However, the observed differences in 6MWD may have been influenced by one or more outliers; sensitivity analyses exploring earlier time points or adjusting for outlier data did not confirm statistically significant differences.

To help contextualise the results of the NMA, it is useful to consider studies of minimum clinically important differences (MCIDs) i.e. the smallest change that patients would notice and consider important. Claeys et al., using data from the PROPEL trial (*n* = 123), found that within-group MCIDs for 6MWD (both % predicted and in metres) depend on the method used (to calculate MCIDs) and on aspects of disease severity (baseline 6MWD, BMI and comorbidities). For their overall population the MCIDs ranged from 24 m to 57 m (2.3–8.1% for % predicted). They concluded that using a single MCID for all patients can be misleading, so a range should be considered [65]. Lika et al. used data from two prospective Dutch studies (*n* = 102) to estimate both between-group and within-group MCIDs [66]. They also reported a range of MCIDs, depending on the methodology used. For the most appropriate anchor methods, the between-group MCIDs ranged from 2.5 to 4.8% for FVC % predicted and from 0.4 to 7.5% for 6MWD % predicted. The within-group MCID was slightly lower (than the between-group MCID) for FVC % predicted and higher for 6MWD.

The comparison of cipaglucosidase alfa with miglustat is complicated by the paucity of direct comparative data in ERT-naïve populations. A recent indirect comparison, using IPD from the PROPEL study and applying multilevel network meta-regression (ML-NMR) analysis to adjust for population differences, suggested cipaglucosidase alfa with miglustat may be more effective than both alglucosidase alfa and avalglucosidase alfa [12]. However, it is unclear whether the ML-NMR methodology adequately accounts for prior ERT treatment, as the COMET trial included only ERT-naïve patients. Moreover, these findings were heavily influenced by non-randomised evidence. Sensitivity analyses restricted to RCT data supported the superiority of cipaglucosidase alfa with miglustat over alglucosidase alfa but indicated that avalglucosidase alfa may be superior to cipaglucosidase alfa with miglustat. The analysis also did not incorporate relevant evidence from the LOTS trial and therefore did not assess a comparison between cipaglucosidase alfa with miglustat and placebo (BSC). The study was also funded by Amicus Therapeutics (which manufactures cipaglucosidase alfa) and four of the authors were Amicus Therapeutics employees.

The long-term evidence on ERT effectiveness was limited to single-group studies. Findings from trial extensions, which provide data up to two years post-randomisation, suggest that initial improvements observed at earlier time points are generally maintained up to around 12–18 months after which declines are seen. This pattern of results is also broadly reflective of the results of the prospective studies identified in the review, although overall they tended to indicate that benefits lasted a little longer (up to 2–3 years). Beyond 2–3 years the evidence suggests steady, gradual declines in both 6MWD and FVC % predicted over up to 10–15 years. However, these results are limited by the generally small sample sizes and uncertainties about the impact of missing data.

The lack of comparative evidence on the long-term effectiveness of ERT leaves key questions unanswered. It remains unclear how ERTs perform relative to BSC over extended periods and whether the initial benefits of ERT are sustained in the long term. Similarly, the absence of robust comparative evidence makes it impossible to draw conclusions about the relative long-term effectiveness of alternative ERTs. This represents a significant limitation of the current evidence base, particularly given the chronic nature of the disease and the substantial costs associated with treatment.

Evidence on ERT-experienced populations is limited. However, available data suggest potential short-term benefits from switching from alglucosidase alfa to cipaglucosidase alfa with miglustat. It is unclear, though, whether these benefits are durable or whether switching ERT treatment poses any risks e.g. antibody formation.

### Limitations

A major limitation of the NMA was the inability to access IPD from the identified RCTs. This would have enabled the use of more advanced statistical synthesis methods and improved the ability to explore treatment-covariate interactions and heterogeneity across studies, potentially allowing for more nuanced conclusions. The lack of engagement by manufacturers and their decision not to provide data, despite established data-sharing agreements, is difficult to justify. Patient experts and stakeholders involved in the project found this lack of cooperation unacceptable and questioned the commitment of the ERT manufacturers, Sanofi and Amicus Therapeutics, to improving patient outcomes.

Additionally, the evidence identified in the review had notable limitations. Two RCTs, PROPEL and LOTS, were assessed as having a high risk of bias due to selective reporting of findings. This raises concerns about the reliability of the estimates for 6MWD and FVC% predicted and whether the treatment effects observed for these outcomes align with unreported results. Although some baseline characteristics were inconsistently reported across the three trials, we nevertheless consider the similarity (transitivity) assumption to be valid for the network, given there is little evidence to suggest any ERT effect modification, based on the subgroup analyses reported in LOTS and COMET.

A further limitation of the RCT evidence is evidence of significant heterogeneity in the effect of alglucosidase alfa. In COMET, alglucosidase showed minimal efficacy in improving 6MWD, contrasting sharply with the positive results observed in the LOTS trial. The EMA highlighted this issue in its evaluation of avalglucosidase, noting concerns about alglucosidase alfa’s underperformance in COMET relative to LOTS. The sponsor attributed the difference to the time gap between the trials, with LOTS conducted when no treatment was available, potentially leading to differences in patient baseline characteristics [67]. While post-hoc analyses were conducted, they have not been fully reported, and the EMA concluded that population differences likely influenced the COMET results. However, this review has identified further data from regulatory documents which indicate skewing of the 6MWD data by outliers, with the means and medians differing substantially; this appears to be a key driver of the variation in the effectiveness of alglucosidase across the published trials (which reported results only as mean values).

Although our review was limited by including only studies written in English, meaning it is possible that relevant studies were missed, it appears unlikely that a small number of additional studies would have meaningfully changed the review’s conclusions, given the consistency of results across studies overall.

### Research recommendations

Our NMA results indicated that long-term, randomised, head-to-head comparisons of ERTs are needed to identify whether there are any meaningful differences in efficacy between ERTs. Studies are also needed to evaluate alternative ERTs in ERT-experienced patients, to better understand the benefits and risks associated with starting a second-line ERT. This will allow clear clinical guidance to be devised to determine appropriate scenarios for switching therapies.

Future studies should also evaluate patient-centred outcomes. The current paucity of comparative data on outcome measures such as the use of mobility aids, need for respiratory support and HRQoL is concerning; our project stakeholders also questioned the appropriateness and relevance of 6MWD and FVC% predicted as meaningful indicators of clinical benefit and highlighted the importance of evaluating the impact of ERT on fatigue. Although many studies use the 6MWD as an outcome measure it has limitations. Bembi et al.’s [39] and Ravaglia et al.’s [50] small studies reported both summary and IPD for 6MWD. These highlighted the wide variation in 6MWD baseline values and responses; in Bembi et al.’s study many patients with low baseline distances had just had a tracheostomy [39]. Ravaglia et al. noted that performance on the 6MWD does not exclusively depend on motor function, but is also affected by respiratory function [50]. The variability of the data in these studies, and in the COMET trial (where there were large differences between mean and median values), illustrate the limitations of the 6MWD as a summary outcome measure for Pompe disease cohorts. Such variation, coupled with inevitably small study sample sizes, means that the reporting of median changes from baseline (in addition to mean changes) should be encouraged in future studies. The 6MWD can also vary within individuals from one measurement to the next; the use of more than one assessment at each time point should therefore be encouraged.

Given the difficulties we had in obtaining IPD, we recommend that sponsors who have signed up to data-sharing platforms show more willingness to share their data with external research teams for independent reviews.

## Conclusions

Our NMA results indicated that ERTs lead to modest improvements in 6MWD and FVC % after one year compared to placebo (BSC without ERT) in ERT-naive populations. However, there is limited evidence to support meaningful differences in outcomes between ERTs. Although longer-term observational data suggest gradual declines in 6MWD and FVC % predicted after two to three years, extending up to at least 15 years, there is a lack of longer-term follow-up data supporting the comparative effectiveness of ERTs. Consequently, there is no clear evidence regarding the extent to which any comparative benefits of ERT relative to placebo (and BSC) are retained in the long term or whether ERT alters the course of Pompe disease, potentially reducing the need for supportive care measures such as walking aids or ventilation.

## Supplementary Information

Below is the link to the electronic supplementary material.

Supplementary Material 1

Supplementary Material 2

Supplementary Material 3

## Data Availability

Not applicable.

## Abbreviations

6-MWD: 6-minute walk test distance
ALG: Alglucosidase alfa
AVAL: Avalglucosidase alfa
BSC: Best supportive care
CM: Cipaglucosidase alfa with miglustat
CENTRAL: Cochrane Central Register of Controlled Trials
CDSR: Cochrane Database of Systematic Reviews
RoB: Cochrane Risk of Bias tool
EMA: European Medicines Agency
ERT: Enzyme replacement therapy
FVC: Forced vital capacity
GAA: Acid α-glucosidase
HHD: Handheld dynamometry
HRQoL: Health-related quality of life
IPD: Individual participant data
IOPD: Infantile onset Pompe disease
LOPD: Late-onset Pompe disease
MCMC: Markov Chain Monte Carlo
MRC: Medical Research Council
NHS EED, MCID: Minimum clinically important difference
ML-NMR: Multilevel network meta-regression
NHS: Economic Evaluations Database
NICE: National Institute for Health and Care Excellence
NIHR: National Institute for Health and Care Research
NMA: Network meta-analyses
PBO: Placebo
PRISMA: Preferred Reporting Items for Systematic Reviews and Meta-Analyses
QMFT: Quick motor function test
RCT: Randomised controlled trial
WHO ICTRP: WHO International Clinical Trials Registry Platform
