# Supplementary material for: Enzyme replacement therapy for the treatment of late onset Pompe disease: A systematic review and network meta-analysis
Source: Orphanet J Rare Dis. 2025 Aug 21;20:451. doi: 10.1186/s13023-025-03981-0 (PMC12372379; doi:10.1186/s13023-025-03981-0)
Supplement: Supplementary file 1 — Supplementary Material 1 [file 13023_2025_3981_MOESM1_ESM.docx]

**Additional file 1: Search Strategies**

**Ovid MEDLINE(R) ALL**

via Ovid <http://ovidsp.ovid.com/>

Date range searched: 1946 to December 07, 2023

Date searched: 12 December 2023

Records retrieved: 2198

1 glycogen storage disease type II/ (1973)

2 (pompe or pompe's or LOPD or LO-PD).ti,ab. (2476)

3 ((alpha glucosidase* or alpha-glucosidase* or alfa glucosidase* or alfa-glucosidase* or "4-glucosidase*" or "4 glucosidase*" or maltase or gaa) adj2 (deficien* or disease*)).ti,ab. (671)

4 (gsdii or gsd ii or gsd2 or "gsd 2" or gsdtwo or gsd two).ti,ab. (218)

5 generali?ed glycogenos?s.ti,ab. (58)

6 (glycogenos?s adj2 (ii or "2" or two)).ti,ab. (300)

7 (glycogen storage adj2 (disease* or disorder*) adj2 (ii or "2" or two)).ti,ab. (433)

8 or/1-7 (3348)

9 exp animals/ not humans.sh. (5176248)

10 8 not 9 (3177)

11 editorial/ or news/ or exp historical article/ (1288048)

12 10 not 11 (3122)

13 limit 12 to yr="2000-Current" (2384)

14 limit 13 to english language (2205)

15 remove duplicates from 14 (2198)

**Key:**

/ or sh = indexing term (Medical Subject Heading: MeSH)

exp = exploded indexing term (MeSH)

* = truncation

? = wildcard for one additional letter

ti,ab = terms in either title or abstract fields

adj2 = terms within two words of each other (any order)

**Embase**

via Ovid <http://ovidsp.ovid.com/>

Date range searched: 1974 to 2023 December 11

Date searched: 12 December 2023

Records retrieved: 2997

1 glycogen storage disease type 2/ (5151)

2 (pompe or pompe's or LOPD or LO-PD).ti,ab. (4433)

3 ((alpha glucosidase* or alpha-glucosidase* or alfa glucosidase* or alfa-glucosidase* or "4-glucosidase*" or "4 glucosidase*" or maltase or gaa) adj2 (deficien* or disease*)).ti,ab. (1014)

4 (gsdii or gsd ii or gsd2 or "gsd 2" or gsdtwo or gsd two).ti,ab. (331)

5 generali?ed glycogenos?s.ti,ab. (36)

6 (glycogenos?s adj2 (ii or "2" or two)).ti,ab. (382)

7 (glycogen storage adj2 (disease* or disorder*) adj2 (ii or "2" or two)).ti,ab. (640)

8 or/1-7 (6230)

9 (animal/ or animal experiment/ or animal model/ or animal tissue/ or nonhuman/) not exp human/ (6871749)

10 8 not 9 (5847)

11 (letter or editorial or note).pt. (3055463)

12 10 not 11 (5518)

13 (conference abstract* or conference review or conference paper or conference proceeding).db,pt,su. (5771584)

14 12 not 13 (3456)

15 limit 14 to yr="2000-Current" (2796)

16 (conference abstract* or conference review or conference paper or conference proceeding).db,pt,su. (5771584)

17 12 and 16 (2062)

18 limit 17 to yr="2020-Current" (466)

19 15 or 18 (3262)

20 limit 19 to english language (3022)

21 remove duplicates from 20 (2997)

**Key:**

/ or .sh. = indexing term (Emtree Subject Heading)

exp = exploded indexing term (Emtree)

* = truncation

? = wildcard for one additional letter

ti,ab = terms in either title or abstract fields

adj2 = terms within two words of each other (any order)

db = database field

su = source type

pt = publication type

**EB Health - KSR Evidence**

via Ovid <http://ovidsp.ovid.com/>

Date range searched: 2015 to 2023 Week 50

Date searched: 12 December 2023

Records retrieved: 20

1 (pompe or pompe's or LOPD or LO-PD).ti,ab. (19)

2 ((alpha glucosidase* or alpha-glucosidase* or alfa glucosidase* or alfa-glucosidase* or "4-glucosidase*" or "4 glucosidase*" or maltase or gaa) adj2 (deficien* or disease*)).ti,ab. (1)

3 (gsdii or gsd ii or gsd2 or "gsd 2" or gsdtwo or gsd two).ti,ab. (0)

4 generali?ed glycogenos?s.ti,ab. (0)

5 (glycogenos?s adj2 (ii or "2" or two)).ti,ab. (0)

6 (glycogen storage adj2 (disease* or disorder*) adj2 (ii or "2" or two)).ti,ab. (0)

7 or/1-6 (20)

8 limit 7 to yr="2000 -Current" (20)

9 remove duplicates from 8 (20)

**Key:**

* = truncation

? = wildcard for one additional letter

ti,ab = terms in either title or abstract fields

adj2 = terms within two words of each other (any order)

**EconLit**

via Ovid <http://ovidsp.ovid.com/>

Date range searched: 1886 to November 23, 2023

Date searched: 12 December 2023

Records retrieved: 4

1 (pompe or pompe's or LOPD or LO-PD).ti,ab. (5)

2 ((alpha glucosidase* or alpha-glucosidase* or alfa glucosidase* or alfa-glucosidase* or "4-glucosidase*" or "4 glucosidase*" or maltase or gaa) adj2 (deficien* or disease*)).ti,ab. (0)

3 (gsdii or gsd ii or gsd2 or "gsd 2" or gsdtwo or gsd two).ti,ab. (0)

4 generali?ed glycogenos?s.ti,ab. (0)

5 (glycogenos?s adj2 (ii or "2" or two)).ti,ab. (0)

6 (glycogen storage adj2 (disease* or disorder*) adj2 (ii or "2" or two)).ti,ab. (0)

7 or/1-6 (5)

8 limit 7 to yr="2000 -Current" (4)

9 remove duplicates from 8 (4)

**Key:**

* = truncation

? = wildcard for one additional letter

ti,ab = terms in either title or abstract fields

adj2 = terms within two words of each other (any order)

**NHS Economic Evaluations Database (NHS EED)**

via https://www.crd.york.ac.uk/CRDWeb/

Date range searched: Inception to 31^st^ March 2015.

Date searched: 12 December 2023

Records retrieved: 10

1 MeSH DESCRIPTOR glycogen storage disease type II IN NHSEED 2

2 (pompe* or LOPD or LO-PD) IN NHSEED 12

3 ((alpha glucosidase* or alpha-glucosidase* or alfa glucosidase* or alfa-glucosidase* or "4-glucosidase*" or "4 glucosidase*" or maltase or gaa) NEAR2 (deficien* or disease*)) IN NHSEED 0

4 (gsdii or gsd ii or gsd2 or "gsd 2" or gsdtwo or gsd two) IN NHSEED 0

5 (generali* NEAR2 glycogenos*) IN NHSEED 0

6 (glycogenos* NEAR2 (ii or "2" or two)) IN NHSEED 0

7 (glycogen storage NEAR2 (disease* or disorder*) NEAR2 (ii or "2" or two)) IN NHSEED 2

8 #1 OR #2 OR #3 OR #4 OR #5 OR #6 OR #7 12

9 (#8) IN NHSEED FROM 2000 TO 2015 10

**Key:**

MeSH Description = indexing term (Medical Subject Heading: MeSH)

* = truncation

NEAR2 = terms within two words of each other

**Cochrane Database of Systematic Reviews (CDSR)**

via Wiley <http://onlinelibrary.wiley.com/>

Issue 12 of 12, December 2023

Date searched: 12 December 2023

Records retrieved: 65

#1 [mh ^"glycogen storage disease type II"] 47

#2 (pompe or pompe's or LOPD or "LO-PD"):ti,ab 213

#3 ((alpha NEXT glucosidase* or alfa NEXT glucosidase* or 4 NEXT glucosidase* or maltase or gaa) NEAR/2 (deficien* or disease*)):ti,ab 21

#4 (gsdii or "gsd ii" or gsd2 or "gsd 2" or gsdtwo or "gsd two"):ti,ab 11

#5 generali?ed glycogenos?s:ti,ab 0

#6 (glycogenos?s NEAR/2 (ii or "2" or two)):ti,ab 3

#7 (glycogen NEXT storage NEAR/2 (disease* or disorder*) NEAR/2 (ii or "2" or two)):ti,ab 14

#8 {OR #1-#7} with Cochrane Library publication date Between Jan 2000 and Dec 2023, in Cochrane Reviews 65

**Key:**

mh ^ = unexploded subject heading (MeSH heading)

* = truncation

? = wildcard for one additional letter

ti,ab = terms in title or abstract fields

near/3 = terms within three words of each other

next = terms are next to each other

**Cochrane Central Register of Controlled Trials (CENTRAL)**

via Wiley <http://onlinelibrary.wiley.com/>

Issue 11 of 12, November 2023

Date searched: 12 December 2023

Records retrieved: 153

#1 [mh ^"glycogen storage disease type II"] 47

#2 (pompe or pompe's or LOPD or "LO-PD"):ti,ab 213

#3 ((alpha NEXT glucosidase* or alfa NEXT glucosidase* or 4 NEXT glucosidase* or maltase or gaa) NEAR/2 (deficien* or disease*)):ti,ab 21

#4 (gsdii or "gsd ii" or gsd2 or "gsd 2" or gsdtwo or "gsd two"):ti,ab 11

#5 generali?ed glycogenos?s:ti,ab 0

#6 (glycogenos?s NEAR/2 (ii or "2" or two)):ti,ab 3

#7 (glycogen NEXT storage NEAR/2 (disease* or disorder*) NEAR/2 (ii or "2" or two)):ti,ab 14

#8 {OR #1-#7} with Publication Year from 2000 to 2023, in Trials 153

**Key:**

mh ^ = unexploded subject heading (MeSH heading)

* = truncation

? = wildcard for one additional letter

ti,ab = terms in title or abstract fields

near/3 = terms within three words of each other

next = terms are next to each other

**International HTA database**

via <https://database.inahta.org/>

Date range searched: Inception – 12 December 2023

Date searched: 12 December 2023

Records retrieved: 11

((((glycogen storage and (disease* or disorder*) and (ii or "2" or two)))[Title] OR ((glycogen storage and (disease* or disorder*) and (ii or "2" or two)))[abs]) OR (((glycogenos* and (ii or "2" or two)))[Title] OR ((glycogenos* and (ii or "2" or two)))[abs]) OR ((generali* and glycogenos*)[Title] OR (generali* and glycogenos*)[abs]) OR ((((alpha glucosidase* or alpha-glucosidase* or alfa glucosidase* or alfa-glucosidase* or "4-glucosidase*" or "4 glucosidase*" or maltase or gaa) and (deficien* or disease*)))[Title] OR (((alpha glucosidase* or alpha-glucosidase* or alfa glucosidase* or alfa-glucosidase* or "4-glucosidase*" or "4 glucosidase*" or maltase or gaa) and (deficien* or disease*)))[abs]) OR (((pompe or pompe's or LOPD or LO-PD))[Title] OR ((pompe or pompe's or LOPD or LO-PD))[abs]) OR (("Glycogen Storage Disease Type II"[mh])))

FROM 2000 TO 2023 = 11

**Key:**

[mh] = indexing term: Medical Subject Heading (MeSH)

[abs] = search of abstract field

[Title] = search of title field

* = truncation

**ClinicalTrials.gov**

via <https://clinicaltrials.gov/>

Date searched: 12 December 2023

Records retrieved: 489

Condition or disease: pompe

= 140

Condition or disease: (glucosidase OR maltase OR gaa) AND (deficiency OR disease)

= 170

Condition or disease: ((glycogen OR GSD) AND (ii OR 2 OR two))

= 179

**European Union Clinical Trials Register**

via [www.clinicaltrialsregister.eu/ctr-search/search](http://www.clinicaltrialsregister.eu/ctr-search/search)

Date searched: 12 December 2023

Records retrieved: 280

Advanced Search: pompe*

= 83

Advanced Search: (glucosidase* OR maltase OR gaa) AND (deficien* OR disease*)

= 133

Advanced Search: ((glycogen* OR GSD) AND (ii OR 2 OR two))

= 64

**WHO ICTRP**

via <https://trialsearch.who.int/>

Date searched: 12 December 2023

Records retrieved: 262

Condition: pompe*

Recruitment Status: All

= 154

Condition: (glucosidase* OR maltase OR gaa) AND (deficien* OR disease*)

Recruitment Status: All

= 37

Condition: ((glycogen* OR GSD) AND (ii OR 2 OR two))

Recruitment Status: All

= 71
