# Supplementary material for: Enzyme replacement therapy for the treatment of late onset Pompe disease: A systematic review and network meta-analysis
Source: Orphanet J Rare Dis. 2025 Aug 21;20:451. doi: 10.1186/s13023-025-03981-0 (PMC12372379; doi:10.1186/s13023-025-03981-0)
Supplement: Supplementary file 2 — Supplementary Material 2 [file 13023_2025_3981_MOESM2_ESM.docx]

**Additional File 2 – RCT, NMA and RCT extension study details**

**Table 1: RCTs risk of bias assessment results (using August 2019 template)**

| **Bias domains and signalling questions** | **Responses, supporting text and judgements** | | |
| --- | --- | --- | --- |
|  | **LOTS(1)** | **COMET(2, 4)** | **PROPEL(3)** |
| ***Randomisation process*** | | | |
| 1.1 Was the allocation sequence random? | Y: Randomisation was performed using a centralised Interactive Voice Response System | Y: A centralised treatment allocation system (interactive response technology) was used. | Y: Patients were randomly assigned using proprietary and validated interactive response technology software |
| 1.2 Was the allocation sequence concealed? | Y: See 1.1 | Y: See 1.1 | Y: See 1.1 |
| 1.3 Were there baseline imbalances that suggest a problem with the randomization process? | No: although there were notable imbalances in age at onset, sex, and use of a walking device which were likely due to chance | No: although chance imbalances in age and age at onset | N |
| **Risk of bias judgement** | LOW (although chance imbalances exist in sex, age at symptom onset and use of walking device) | LOW | LOW |
| ***Deviations from the intended interventions*** | | | |
| 2.1. Were participants aware of their assigned intervention during the trial? | PN: The trial was placebo-controlled though details of blinding were not reported. EMA assessment report was reassuring on this issue. | PN: The two treatments have the same dosing schedule and administration method. EMA assessment report states that “The plans described in relation to blinding measures are generally considered adequate” | PN: Cipaglucosidase alfa and alglucosidase were both administered intravenously and at the same frequency. A matching placebo for miglustat was used with alglucosidase alfa, and black or dark covering over cipaglucosidase alfa and alglucosidase alfa reconstituted solution were used during infusion. |
| 2.2. Were carers and people delivering the interventions aware of participants’ assigned intervention during the trial? | PN: see 2.1 | PN: see 2.1 | PN: See 2.1 |
| 2.3. If Y/PY/NI to 2.1 or 2.2: Were there deviations from the intended interventions that arose because of the trial context? | NA | NA | NA |
| 2.4 If Y/PY to 2.3: Were these deviations likely to have affected the outcome? | NA | NA | NA |
| 2.5 If Y/PY/NI to 2.4: Were these deviations from intended intervention balanced between groups? | NA | NA | NA |
| 2.6 Was an appropriate analysis used to estimate the effect of assignment to intervention? | NA | NA | NA |
| 2.7 If N/PN/NI to 2.6: Was there potential for a substantial impact (on the result) of the failure to analyse participants in the group to which they were randomized? | NA | NA | NA |
| **Risk of bias judgement** | LOW | LOW | LOW |
| ***Missing outcome data*** | | | |
| 3.1 Were outcome data available for all, or nearly all, participants randomised? | N: 90% completed the study | Y: 95% of participants completed the primary analysis period | Y: 5% discontinued treatment. 2 Alg + placebo patients were not dosed due to absence of genotype confirmation |
| 3.2 If N/PN/NI to 3.1: Is there evidence that the  result was not biased by missing outcome data? | N | NA | NA |
| 3.3 If N/PN to 3.2: Could missingness in the outcome depend on its true value? | Y | NA | NA |
| 3.4 If Y/PY/NI to 3.3: Is it likely that missingness in the outcome depended on its true value? | N: 5% difference in drop-outs between groups. Reasons for missing data were similar. | NA | NA |
| **Risk of bias judgement** | SOME CONCERNS | LOW | LOW |
| ***Measurement of the outcome*** | | | |
| 4.1 Was the method of measuring the outcome inappropriate? | N | N | N |
| 4.2 Could measurement or ascertainment of the outcome have differed between intervention groups? | N | N | Y: Covid 19 resulted in some patients experiencing missed infusions, missed assessments or delayed visits. Delayed visits and makeup assessments were allowed, and the data were assigned to a specified visit timepoint (for the analyses) when appropriate, i.e. data from the delayed visits were remapped to the earlier planned study visits. The EMA noted that using an analysis model based on *actual* (rather than remapped) time points is expected to lead to a more reliable estimation of the treatment difference. The EMA reported that there were 17 subjects (13 vs. 4) with a delayed visit of at least 4 weeks after the target day, 8 of these 17 subjects (8 vs. 0) had a delay of at least 6 weeks after the target day.(13) See 5.3 below. |
| 4.3 If N/PN/NI to 4.1 and 4.2: Were outcome assessors aware of the intervention received by study participants? | N | N | N |
| 4.4 If Y/PY/NI to 4.3: Could assessment of the outcome have been influenced by knowledge of intervention received? | NA | NA | NA |
| 4.5 If Y/PY/NI to 4.4: Is it likely that assessment of the outcome was influenced by knowledge of intervention received? | NA | NA | NA |
| **Risk of bias judgement** | LOW | LOW | LOW |
| ***Selection of the reported result*** | | | |
| 5.1 Were the data that produced this result analysed in accordance with a pre-specified analysis plan that was finalized before unblinded outcome data were available for analysis? | Y, though not all results were reported (see below) | Y: Analyses were planned using a mixed model for repeated measures (MMRM) using change from baseline; treatment difference estimated based on least square means at week 49. The EMA considered this to be adequate. | Y: Primary endpoint analysis (6MWD) used a mixed effect model with repeated measures using observed values. Secondary endpoints were analysed using an ANCOVA model. For sensitivity analyses, ANCOVA was used for 6MWD and MMRM was used for all key secondary efficacy endpoints. |
| Is the numerical result being assessed likely to have been selected, on the basis of the results, from...  5.2. ... multiple eligible outcome measurements (e.g. scales, definitions, time points) within the outcome domain? | N | N | N |
| 5.3 ... multiple eligible analyses of the data? | Y: From EMA report - Analyses were planned using a LME model. Sensitivity analyses was performed with WMW test and with analysis of covariance. The data did not fit the model assumptions of the LME model. The EMA stated that the ANCOVA model could not be considered as valid since the analysis of the residuals distribution was lacking, but that the WMW results could be assessed since its validity was less questionable.(14)The published paper presented results based on the ANCOVA analyses. | N (see 5.1) | Y: The paper did not report all the analyses (see 5.1 above) for all key secondary outcomes, nor for all the key outcomes for the ERT naive/experienced subgroups.  The company thought that the normality assumption for the pre-defined MMRM analysis was significantly violated and the non-parametric ANCOVA results were more appropriate. The EMA disagreed, noting that i) visual inspection of the residuals from both the original and the (EMA) requested MMRM analyses did not suggest violation of the normality assumption, and ii) the ANCOVA analyses used LOCF for handling missing data and was performed based on remapping of visit time points (not the actual time points – see 4.2 above); EMA thought that both could introduce bias in the estimated efficacy treatment difference.(13) |
| **Risk of bias judgement** | HIGH | LOW | HIGH |
| ***Overall risk of bias*** | HIGH | LOW | HIGH |

EMA European Medicines Agency, LME linear mixed effects, LOCF Last observation carried forward, N No, NA Not applicable, WMW Wilcoxon-Mann-Whitney, Y Yes

1. **Summary characteristics of included RCTs**

Baseline characteristics such as age at disease onset, age at diagnosis, age at start of ERT, percentage of male patients, percentage of patients needing respiratory support (on ventilator), percentage of patients on wheelchair (ambulatory) were extracted from the studies where they were reported. The baseline characteristics of the population in each RCT is presented in ***Table***  below. LOTS and COMET recruited ERT-naïve patients in their studies but PROPEL recruited both ERT- experience and ERT naïve patients. However, the NMA used results from the ERT naïve population.

**Table 2: Baseline characteristics of RCT cohorts**

| **Study** | **Treatment group (N)** | **Mean age**  **Mean (SD)** | **% Male** | **Mean age at onset^1^ (SD)** | **% ERT Naive** | **Disease duration (years)** | **% c-32-13T>G**  **mutation** | **% Use of walking aid** | **Mean 6MWD, m (SD)** | **Mean 6MWD % Predicted (SD)** | **Mean FVC**  **% predicted (SD)** |
| --- | --- | --- | --- | --- | --- | --- | --- | --- | --- | --- | --- |
| **LOTS** **(1)** | ALG (60) | 45.3 (12.4) | 57 | 30.3 (12.3) | 100 | 9.0 (6.3) | NR | 38 | 332.2 (126.7) | 52.5 (19.0) | 55.4 (14.4) |
|  | PLB (30) | 42.6 (11.6) | 37 | 23.9 (11.0) | 100 | 10.1 (8.4) | NR | 53 | 317.9 (132.3) | 50.3 (20.5) | 53.0 (15.7) |
|  | Total (90) | 44.4 (16.98) ^2^ | 50 | 28.2 (16.5) ^2^ | 100 | 9.37 (10.5) ^2^ | NR | 43 | 327.4 (183.2) ^2^ | 51.8 (28.0) ^2^ | 54.6 (21.3) ^2^ |
| **COMET (2)** | AVAL (51) | 46 (14.5) | 53 | 32.9 (16.6) ^3^ | 100 | NR | 84 | NR | 399.3 (110.9) | 57.3 (15.0) | 62.5 (14.4) |
|  | ALG (49) | 50.3 (13.7) | 51 | 37.7 (15.7) | 100 | NR | 94 | NR | 378.1 (116.2) | 55.3 (16.6) | 61.6 (12.4) |
|  | Total (100) | 48.1 (14.2) | 52 | 35.3 (16.3) ^4^ | 100 | NR | 89 | NR | 388.9 (113.5) | 56.3 (15.8) | 62.1 (13.4) |
| **PROPEL** **(3)** | CM (85)  Naïve (20)^5^ | 47.6 (13.3) | 42 | NR | 24 | NR | 89 | 20 | 357.9 (111.8)  393.6 (112.4) ^5^ | NR  61.9 (15.3) ^5^ | 70.7 (19.6)  80.2 (18.7) ^5^ |
|  | ALG (38)  Naïve (7) ^5^ | 45.1 (13.3) | 53 | NR | 21 | NR | 84 | 29 | 351.0 (121.3)  420.9 (135.7) ^5^ | NR  61.4 (17.1) ^5^ | 69.7 (21.5)  79.1 (22.6) ^5^ |
|  | Total (123) | 46.8 (18.8) ^2^ | 46 | NR | 23 | NR | 88 | 23 | 355.8 (165.0) ^2^ | NR | 70.4 (29.1) ^2^ |
| **Notes:**  ^1^ At first symptoms  ^2^ implies that the values were calculated assuming both treatment groups are independent  ^3^ Based on 50 participants in AVAL  ^4^ Based on 99 participants  ^5^ERT naive population  **Abbreviations:** ALG Alglucosidase; AVAL Avalglucosidase; PLB placebo; CM Cipaglucosidase + Miglustat; NR Not reported; SD, standard deviation | | | | | | | | | | | |

1. **RCT results for the Network Meta-analyses (NMA)**

The extracted outcomes used in the NMA were 6MWD (m) and percentage (%) force vital capacity (FVC) because these two outcomes were reported by all three RCTs (LOTS, COMET and PROPEL).

**Table 3: Results of key review outcomes from LOTS, COMET and PROPEL (ERT naïve population)**

| **Outcome** | **LOTS (1)** | | | **COMET (2), (4)** | | | **PROPEL (3) (ERT naïve population)** | | |
| --- | --- | --- | --- | --- | --- | --- | --- | --- | --- |
|  | Change from baseline at  week 78 (SE) | | Difference in means (95% CI) | Change from baseline at  week 49 (SE) | | Difference in means (95% CI) | Change from baseline at  week 52 (SE) | | LS mean Difference (95% CI)^2^ |
|  | ALG | PLB |  | AVAL | ALG |  | CM | ALG+PLB |  |
| **Motor function** | | | | | | | | | |
| 6MWD (m) | 25.13 (7.68) ^1^ | -2.99 (10.80) ^1^ | 28.12 (2.07, 54.17) | 32.21 (9.93) | 2.19 (10.40) | 30.01 (1.33, 58.69) | 33.4 (48.7) | 38.3 (29.3) | -9.0 (-46.5, 35.0) |
| 6MWD, % predicted | NR | NR | NR | 5.02 (1.54) | 0.31 (1.62) | 4.71 (0.25, 9.17) | 6.9 (1.8) | 7.2 (1.7) | -4.9 (-12.6, 2.8) |
| **Pulmonary function** | | | | | | | | | |
| FVC % predicted | 1.20 (0.70) ^1^ | -2.20 (0.98) ^1^ | 3.40 (1.03, 5.77) | 2.89 (0.88) | 0.46 (0.93) | 2.43 (-0.13, 4.99) | -4.1 (1.5) | -3.6 (1.8) | -2.0 (-8.9, 5.0) |
| **Notes:**  ^1^ SE calculated using the reported 95% CI  ^2^ Least Square mean is reported  **Abbreviations:** ALG Alglucosidase; AVAL Avalglucosidase; PLB placebo; CM Cipaglucosidase + Miglustat; NR Not reported; CI confidence interval; FVC forced vital capacity; PLB is placebo; 6MWT Six-minute Walk time; m metres; SE, standard error.; LS least square. | | | | | | | | | |

1. **R-code for network meta-analysis**.

The NMA (Bayesian framework) was employed to estimate the indirect treatment effectiveness of the therapies evaluated in the RCTs. This was carried using the Rjags package in R Studio software.(5)The code is presented below:

### call up the packages needed

library(dplyr)

library(rjags)

library(coda)

library(R2jags)

## load the dataset (called d_mwd in this analysis) and prepare data set to be used in the jags model

## nt = number of treatments; ns = number of studies; t1 = reference treatment in each RCT; t2 = interventional treatment in each RCT

d.jags<- list(ns=3, nt =4, y= d_mwd$y, se = d_mwd$se, t1 = d_mwd$t1, t2 = d_mwd$t2)

i.jags<- function(){list(d=c(NA,0,0,0))}

p.jags <- c("d","diff", "totresdev")

## Model adapted from the Technical Support Document (TSD) 2(6) written as nma_RE.txt

Model {

for(i in 1:ns) {

y[i] ~ dnorm(delta[i],prec[i])

prec[i]<- pow(se[i],-2)

dev[i] <- (y[i]-delta[i])*(y[i]-delta[i])*prec[i]

delta[i] ~ dnorm(md[i],tau)

md[i] <- d[t2[i]] - d[t1[i]] # mean of treat effects distributions

}

totresdev <- sum(dev[]) #Total Residual Deviance

d[1]<-0 # treatment effect is zero for reference treatment

for (k in 2:nt){ d[k] ~ dnorm(0,.0001) } # vague priors for treatment effects

sd ~ dunif(0,5) # vague prior for between-trial SD

tau <- pow(sd,-2) # between-trial precision = (1/between-trial variance)

# All pairwise comparisons

for (c in 1:(nt-1)){for (k in (c+1):nt){diff[k,c]<- d[k] - d[c]}}

}

### run the analysis

m <- jags.model(d.jags, file = "nma_RE.txt",inits = i.jags,n.chains = 3)

update(m, 5000)

res <- coda.samples(m, variable.names = p.jags, n.iter = 500000, thin = 20)

summary(res)

## traceplot

Plot(res)

1. **Digitization of KM from RCTs included in the NMA**

The changes from baseline values (Mean and 95% CI) for 6MWD and FVC of the treatments evaluated in each RCT at varying timepoints were extracted by digitizing the change in baseline curves using the plotDiogitizer software on the website (https://plotdigitizer.com/). The digitised values is reported in the ***Table 4*** and ***Table*** below. The digitized values were use in the NMA primary, sensitivity and explorative analysis.

**Table 4: Digitized values from the change in baseline 6MWD**

| **6MWD (m)** | **LOTS** | | | **COMET** | | | **PROPEL** | | |
| --- | --- | --- | --- | --- | --- | --- | --- | --- | --- |
|  | **Alglu** | **Placebo** | **Difference** | **Aval** | **Alglu** | **Difference** | **Cipa + Mig** | **Algu** | **Difference** |
| **12 / 13 weeks** | 14.42 (8.59 to 20.26) | -1.79 (-11.13 to 7.54) | 16.22 (5.2 to 27.23) | 18.06 (1.06 to 35.07) | 15.03 (-2.73 to 32.8) | 3.03 (-21.56 to 27.63) | 17.16 (7.69 to 26.63) | 20.48 (4.23 to 36.74) | -3.32 (-22.13 to 15.5) |
| **25 / 26 weeks** | 27.88 (17.42 to 38.34) | 3.64 (-7.09 to 14.37) | 24.24 (9.26 to 39.22) | 27.23 (8.01 to 46.45) | 9.55 (-10.81 to 29.91) | 17.68 (-10.32 to 45.68) | 32.16 (14.5 to 49.83) | 30.43 (16.3 to 44.57) | 1.73 (-20.9 to 24.36) |
| **37/ 38 weeks** | 29.3 (15.9 to 42.7) | -0.04 (-13.31 to 13.23) | 29.34 (10.48 to 48.2) | 28.52 (11 to 46.03) | 15.42 (-2.85 to 33.69) | 13.1 (-12.21 to 38.41) | 25.24 (4.89 to 45.59) | 38.65 (24.38 to 52.93) | -13.41 (-38.27 to 11.45) |
| **49 / 52 weeks** | 27.04 (12.97 to 41.12) | 1.22 (-13.86 to 16.29) | 25.83 (5.21 to 46.45) | 32.13 (12.85 to 51.41) | 2.06 (-16.52 to 20.65) | 30.06 (3.28 to 56.85) | 33.44 (12 to 54.89) | 38.3 (16.53 to 60.07) | -4.86 (-35.41 to 25.7) |
| **Last follow-up ^** | 28.13 (10.36 to 45.91) | -2.38 (-21.87 to 17.12) | 30.51 (4.13 to 56.89) | 32.13 (12.85 to 51.41) | 2.06 (-16.52 to 20.65) | 30.06 (3.28 to 56.85) | 33.44 (12 to 54.89) | 38.3 (16.53 to 60.07) | -4.86 (-35.41 to 25.7) |
| **Note:** Last follow-up during the double-blind phase of the RCT; week 78 in LOTS trial, week 49 in the COMET trial and week 52 in the PROPEL trial | | | | | | | | | |

**Table 5: Digitized values from the change in baseline FVC**

| **FVC (% predicted)** | **LOTS** | | | **COMET** | | | **PROPEL** | | |
| --- | --- | --- | --- | --- | --- | --- | --- | --- | --- |
|  | **Alglu** | **Placebo** | **Difference** | **Aval** | **Alglu** | **Difference** | **Cipa + Mig** | **Algu** | **Difference** |
| **12 / 13 weeks** | 1.8 (0.83 to 2.77) | -0.82 (-2.25 to 0.62) | 2.61 (0.86 to 4.37) | 3.04 (1.54 to 4.53) | 0.64 (-0.91 to 2.19) | 2.4 (0.24 to 4.55) | 0.27 (-2.01 to 2.55) | -0.95 (-4.11 to 2.21) | 1.22 (-2.68 to 5.12) |
| **25 / 26 weeks** | 1.59 (0.47 to 2.7) | -0.33 (-1.59 to 0.94) | 1.91 (0.21 to 3.62) | 3.21 (1.67 to 4.74) | 0.57 (-1.04 to 2.19) | 2.63 (0.41 to 4.86) | -1.51 (-4.17 to 1.14) | -4.4 (-8.3 to -0.49) | 2.89 (-1.84 to 7.61) |
| **37/ 38 weeks** | 1.35 (0.09 to 2.6) | -2.45 (-4.08 to -0.82) | 3.8 (1.7 to 5.91) | 2.2 (0.25 to 4.15) | 0.55 (-1.45 to 2.55) | 1.65 (-1.14 to 4.44) | -4.08 (-6.33 to -1.83) | -0.58 (-7.1 to 5.93) | -3.5 (-10.39 to 3.4) |
| **49 / 52 weeks** | 1.65 (0.35 to 2.96) | -1.95 (-3.7 to -0.2) | 3.6 (1.4 to 5.8) | 2.89 (1.19 to 4.58) | 0.46 (-1.33 to 2.24) | 2.43 (-0.03 to 4.89) | -4.1 (-7 to -1.21) | -3.64 (-7.14 to -0.14) | -0.46 (-5.01 to 4.08) |
| **Last follow-up** | 1.32 (-0.2 to 2.85) | -2.61 (-4.41 to -0.81) | 3.93 (1.55 to 6.32) | 2.89 (1.19 to 4.58) | 0.46 (-1.33 to 2.24) | 2.43 (-0.03 to 4.89) | -4.1 (-7 to -1.21) | -3.64 (-7.14 to -0.14) | -0.46 (-5.01 to 4.08) |
| **Note:** Last follow-up during the double-blind phase of the RCT; week 78 in LOTS trial, week 49 in the COMET trial and week 52 in the PROPEL trial | | | | | | | | | |

1. **Results of NMA**

This section presents all the estimates obtained from the NMA (both random effects and fixed effects models). The estimates are presented as mean difference (95% credible interval).

1. ***Results of primary analysis***

The primary analysis evaluated the treatment effectiveness of AVAL, ALG, CM, and placebo using digitized data from 49/52 weeks for all the RCTS (LOTS, PROPEL and COMET) in ERT naïve population. NMA was carried out using FE am RE models. The results for the RE is presented in the main article and the result from the FE is presented below (**Table 6**).

**Table 6: Primary analysis – relative treatment effects of FVC and 6MWD (RE and FE models)**

| **Mean differences (95% credible interval) from random effects NMA** | | | | |
| --- | --- | --- | --- | --- |
| **Outcome: Force vital capacity (FVC)** | | | | |
|  | **PBO** | **ALG** | **AVAL** | **CM** |
| **PBO** |  | 3.58 (-2.95, 10.13) | 6.01 (-3.40, 15.33) | 3.11 (-6.79, 13.00) |
| **ALG** | -3.58 (-10.13, 2.95) |  | 2.43 (-4.22, 9.07) | -0.48 (-7.97, 6.98) |
| **AVAL** | -6.01 (-15.33, 3.40) | -2.43 (-9.07, 4.22) |  | -2.90 (-12.91, 7.07) |
| **CM** | -3.11 (-13.00, 6.79) | 0.48 (-6.98, 7.97) | 2.90 (-7.07, 12.91) |  |
| **Outcome: Six-minute walking distance (6MWD)** | | | | |
|  | **PBO** | **ALG** | **AVAL** | **CM** |
| **PBO** |  | 24.68 (3.97, 45.65) | 53.55 (19.66, 87.31) | 19.29 (-17.43, 56.09) |
| **ALG** | -24.68 (-45.65, -3.97) |  | 28.87 (1.74, 55.66) | -5.39 (-35.72, 25.58) |
| **AVAL** | -53.55 (-87.31, -19.66) | -28.87 (-55.66, -1.74) |  | -34.26 (-74.80, 6.96) |
| **CM** | -19.29 (-56.09, 17.43) | 5.39 (-25.58, 35.72) | 34.26 (-6.96, 74.80) |  |
| **Mean differences (95% credible interval) from fixed effects NMA** | | | | |
| **Outcome: Force vital capacity (FVC)** | | | | |
|  | **PBO** | **ALG** | **AVAL** | **CM** |
| **PBO** |  | 3.60 (1.40, 5.80) | 6.03 (2.74, 9.35) | 3.11 (-1.94, 8.16) |
| **ALG** | -3.60 (-5.80, -1.40) |  | 2.43 (-0.02, 4.87) | -0.49 (-5.04, 4.03) |
| **AVAL** | -6.03 (-9.35, -2.74) | -2.43 (-4.87, 0.02) |  | -2.92 (-8.07, 2.21) |
| **CM** | -3.11 (-8.16, 1.94) | 0.49 (-4.03, 5.04) | 2.92 (-2.21, 8.07) |  |
| **Outcome: Six-minute walking distance (6MWD)** | | | | |
|  | **PBO** | **ALG** | **AVAL** | **CM** |
| **PBO** |  | 24.74 (4.59, 44.93) | 53.71 (20.50, 86.93) | 19.43 (-16.68, 55.44) |
| **ALG** | -24.74 (-44.93, -4.59) |  | 28.97 (2.33, 55.67) | -5.31 (-35.29, 24.81) |
| **AVAL** | -53.71 (-86.93, -20.50) | -28.97 (-55.67, -2.33) |  | -34.28 (-74.37, 5.97) |
| **CM** | -19.43 (-55.44, 16.68) | -5.31 (-24.81, 35.29) | 34.28 (-5.97, 74.37) |  |
| **Notes:** Column treatment versus row treatment  **Abbreviation**: ALG, Alphaglucosidase; AVAL, Avalglucosidase; CM, Cipaglucosidase + Miglustat; PBO, placebo; FVC, forced vital capacity, 6MWD six-minute walking distance; | | | | |

1. ***Results of sensitivity analysis***

The sensitivity analysis evaluated the treatment effectiveness of AVAL, ALG, CM, and placebo using digitized data from 49/52 weeks for LOTS and PROPEL, and 37/38 weeks for COMET in ERT naïve population. The sensitivity analysis was carried out using 37/38 weeks from COMET because of an outlier in the 6MWD which skewed the data making a substantial difference between the means and medians values. The relative treatment effectiveness is reported in Table 7.

**Table 7: Sensitivity analysis – relative treatment effects of FVC and 6MWD (RE and FE models)**

| **Mean differences (95% credible interval) from random effects NMA** | | | | |
| --- | --- | --- | --- | --- |
| **Outcome: Force vital capacity (FVC)** | | | | |
|  | **PBO** | **ALG** | **AVAL** | **CM** |
| **PBO** |  | 3.59 (-3.00, 10.17) | 5.24 (-4.18, 14.64) | 3.14 (-6.77, 13.10) |
| **ALG** | -3.59 (-10.17, 3.00) |  | 1.64 (-5.09, 8.35) | -0.46 (-7.94, 7.04) |
| **AVAL** | -5.24 (-14.64, 4.18) | -1.64 (-8.35, 5.09) |  | -2.10 (-12.17, 7.95) |
| **CM** | -3.14 (-13.10, 6.77) | 0.46 (-7.04, 7.94) | 2.10 (-7.95, 12.17) |  |
| **Outcome: Six-minute walking distance (6MWD)** | | | | |
|  | **PBO** | **ALG** | **AVAL** | **CM** |
| **PBO** |  | 24.79 (3.65, 45.62) | 37.22 (4.08, 70.10) | 19.38 (-17.51, 56.78) |
| **ALG** | -24.79 (-45.62, -3.65) |  | 12.43 (-13.18, 38.07) | -5.41 (-36.06, 25.24) |
| **AVAL** | -37.22 (-70.10, -4.08) | -12.43 (-38.07, 13.18) |  | -17.84 (-57.83, 22.31) |
| **CM** | -19.38 (-56.78, 17.51) | 5.41 (-25.24, -36.06) | 17.84 (-22.31, 57.83) |  |
| **Mean differences (95% credible interval) from fixed effects NMA** | | | | |
| **Outcome: Force vital capacity (FVC)** | | | | |
|  | **PBO** | **ALG** | **AVAL** | **CM** |
| **PBO** |  | 3.59 (1.37, 5.82) | 5.24 (1.69, 8.79) | 3.12 (-1.93, 8.17) |
| **ALG** | -3.59(-5.82, -1.37) |  | 1.65 (-1.12, 4.43) | -0.47 (-4.98, 4.08) |
| **AVAL** | -5.24 (-8.79, -1.69) | -1.65 (-4.43, 1.12) |  | -2.12 (-7.41, 3.22) |
| **CM** | -3.12 (-8.17, 1.93) | 0.47 (-4.08, 4.98) | 2.12 (-3.22, 7.41) |  |
| **Outcome: Six-minute walking distance (6MWD)** | | | | |
|  | **PBO** | **ALG** | **AVAL** | **CM** |
| **PBO** |  | 24.89 (4.67, 45.13) | 37.32 (5.52, 69.21) | 19.55 (-16.65, 55.82) |
| **ALG** | -24.89 (-45.13, -4.67) |  | 12.43 (-12.29, 37.47) | -5.34 (-35.67, 24.86) |
| **AVAL** | -37.32 (-69.21, -5.52) | -12.43 (-37.47, 12.29) |  | -17.77 (-57.14, 21.25) |
| **CM** | -19.55 (-55.82, 16.65) | 5.34 (-24.86, 35.67) | 17.77 (-21.25, 57.14) |  |
| **Notes:** Column treatment versus row treatment  **Abbreviation**: ALG, Alphaglucosidase; AVAL, Avalglucosidase; CM, Cipaglucosidase + Miglustat; PBO, placebo; FVC, forced vital capacity, 6MWD six-minute walking distance; | | | | |

1. ***Results of other explorative analysis using various timepoints of the digitized dataset***

In addition to the primary and sensitivity analysis carried out using the ERT naïve population; NMA was conducted using the digitized dataset from four timepoints (12/13 weeks, 24/26 weeks, 37/38 weeks and LFU) for all the RCTS. The LFU are 49 weeks, 78 weeks and 52 weeks for COMET, LOTS and PROPEL respectively.

**Table 8: Relative treatment effects of FVC and 6MWD (RE models) for varying timepoints**

| **12 / 13 weeks**  **Mean differences (95% credible interval)** | | | | |
| --- | --- | --- | --- | --- |
| **Outcome: Force vital capacity (FVC)** | | | | |
|  | **PBO** | **ALG** | **AVAL** | **CM** |
| **PBO** |  | 1.91 (-4.54, 8.32) | 4.30 (-4.86, 13.47) | 3.12 (-6.52, 12.67) |
| **ALG** | -1.91 (-8.32, 4.54) |  | 2.39 (-4.11, 8.93) | 1.21 (-5.99, 8.32) |
| **AVAL** | -4.30 (-13.47, 4.86) | -2.39 (-8.93, 4.11) |  | -1.18 (-11.00, 8.42) |
| **CM** | -3.12 (-12.67, 6.52) | -1.21 (-8.32, 5.99) | 1.18 (-8.42, 11.00) |  |
| **Outcome: Six-minute walking distance (6MWD)** | | | | |
|  | **PBO** | **ALG** | **AVAL** | **CM** |
| **PBO** |  | 16.00 (3.65, 28.35) | 18.76 (-8.94, 46.44) | 12.49 (-10.62, 35.48) |
| **ALG** | -16.00 (-28.35, -3.65) |  | 2.76 (-22.24, 27.82) | -3.51 (-23.15, 16.01) |
| **AVAL** | -18.76 (-46.44, 8.94) | -2.76 (-27.82, 22.24) |  | -6.27 (-37.98, 25.51) |
| **CM** | -12.49 (-35.48, 10.62) | 3.51 (-16.01, 23.15) | 6.27 (-25.51, 37.98) |  |
| **24/26 weeks**  **Mean differences (95% credible interval)** | | | | |
| **Outcome: Force vital capacity (FVC)** | | | | |
|  | **PBO** | **ALG** | **AVAL** | **CM** |
| **PBO** |  | 3.80 (-2.75, 10.28) | 6.42 (-2.84, 15.52) | 6.67 (-3.24, 16.57) |
| **ALG** | -3.80 (-10.28, 2.75) |  | 2.62 (-3.98, 9.18) | 2.87 (-4.66, 10.41) |
| **AVAL** | -6.42 (-15.52, 2.84) | -2.62 (-9.18, 3.98) |  | 0.25 (-9.74, 10.22) |
| **CM** | -6.67 (-16.57, 3.24) | -2.87 (-10.41, 4.66) | -0.25 (10.22, 9.74) |  |
| **Outcome: Six-minute walking distance (6MWD)** | | | | |
|  | **PBO** | **ALG** | **AVAL** | **CM** |
| **PBO** |  | 23.60 (7.64, 39.43) | 40.34 (7.78, 72.39) | 24.96 (-2.88, 52.99) |
| **ALG** | -23.60 (-39.43, -7.64) |  | 16.74 (-11.76, 44.95) | 1.36 (-21.82, 24.37) |
| **AVAL** | -40.34 (-72.39, -7.78) | -16.74 (-44.95, 11.76) |  | -15.39 (-51.64, 21.33) |
| **CM** | -24.96 (-52.99, 2.88) | -1.36 (-24.37, 21.82) | 15.39 (-21.33, 51.64) |  |
| **37/38 weeks**  **Mean differences (95% credible interval)** | | | | |
| **Outcome: Force vital capacity (FVC)** | | | | |
|  | **PBO** | **ALG** | **AVAL** | **CM** |
| **PBO** |  | 3.60 (-3.02, 10.15) | 5.25 (-4.22, 14.70) | 0.10 (-10.86, 11.18) |
| **ALG** | -3.60 (-10.15, 3.02) |  | 1.65 (-5.12, 8.40) | -3.50 (-12.50, 5.51) |
| **AVAL** | -5.25 (-14.70, 4.22) | -1.65 (-8.40, 5.12) |  | -5.15 (-16.33, 6.07) |
| **CM** | -0.10 (-11.18, 10.86) | 3.50 (-5.51, 12.50) | 5.15 (-6.07, 16.33) |  |
| **Outcome: Six-minute walking distance (6MWD)** | | | | |
|  | **PBO** | **ALG** | **AVAL** | **CM** |
| **PBO** |  | 28.60 (9.30, 47.83) | 41.10 (9.26, 72.88) | 14.98 (-16.72, 46.71) |
| **ALG** | -28.60 (-47.83, -9.30) |  | 12.50 (-13.31, 38.09) | -13.62 (-39.01, 11.87) |
| **AVAL** | -41.10 (-72.88, -9.26) | -12.50 (-38.09, 13.31) |  | -26.12 (-62.14, 10.13) |
| **CM** | -14.98 (-46.71, 16.72) | 13.62 (-11.87, 39.01) | 26.12 (-10.13, 62.14) |  |

| **Last follow up**  **Mean differences (95% credible interval)** | | | | |
| --- | --- | --- | --- | --- |
| **Outcome: Force vital capacity (FVC)** | | | | |
|  | **PBO** | **ALG** | **AVAL** | **CM** |
| **PBO** |  | 3.38 (-3.27, 9.97) | 5.80 (-3.55, 15.03) | 2.86 (-7.41, 13.18) |
| **ALG** | -3.38 (-9.97, 3.27) |  | 2.42 (-4.27, 9.06) | -0.52 (-8.43, 7.38) |
| **AVAL** | -5.80 (-15.03, 3.55) | -2.42 (-9.06, 4.27) |  | -2.94 (-13.28, 7.36) |
| **CM** | -2.86 (-13.18, 7.41) | 0.52 (-7.38, 8.43) | 2.94 (-7.36, 13.28) |  |
| **Outcome: Six-minute walking distance (6MWD)** | | | | |
|  | **PBO** | **ALG** | **AVAL** | **CM** |
| **PBO** |  | 26.41 (0.36, 52.51) | 55.12 (16.65, 94.05) | 20.63 (-24.03, 66.00) |
| **ALG** | -26.41 (-52.51, -0.36) |  | 28.72 (-0.13, 57.91) | -5.78 (-43.84, 32.42) |
| **AVAL** | -55.12 (-94.05, -16.65) | -28.72 (-57.91, 0.13) |  | -34.49 (-82.72, 13.33) |
| **CM** | -20.63 (-66.00, 24.03) | 5.78 (-32.42, 43.84) | 34.49 (-13.33, 82.72) |  |
| **Notes:** Column treatment versus row treatment  **Abbreviation**: ALG, Alphaglucosidase; AVAL, Avalglucosidase; CM, Cipaglucosidase + Miglustat; PBO, placebo; FVC, forced vital capacity, 6MWD six-minute walking distance; | | | | |

**Table 9: Relative treatment effects of FVC and 6MWD (FE models) for varying timepoints.**

| **12 / 13 weeks**  **Mean differences (95% credible interval)** | | | | |
| --- | --- | --- | --- | --- |
| **Outcome: Force vital capacity (FVC)** | | | | |
|  | **PBO** | **ALG** | **AVAL** | **CM** |
| **PBO** |  | 1.92 (0.20, 3.62) | 4.30 (1.54, 7.04) | 3.14 (-1.13, 7.41) |
| **ALG** | -1.92 (-6.62, -0.20) |  | 2.86 (0.24, 4.54) | 1.22 (-2.69, 5.13) |
| **AVAL** | -4.30 (-7.04, -1.54) | -2.86 (-4.54, -0.24) |  | -1.16 (-5.63, 3.31) |
| **CM** | -3.14 (-7.41, 1.13) | -1.22 (-5.13, 2.69) | 1.16 (-3.31, 5.63) |  |
| **Outcome: Six-minute walking distance (6MWD)** | | | | |
|  | **PBO** | **ALG** | **AVAL** | **CM** |
| **PBO** |  | 16.06 (5.05, 26.99) | 18.79 (-7.75, 45.45) | 12.63 (-9.02, 34.10) |
| **ALG** | -16.06 (-26.99, -5.05) |  | 2.73 (-21.60, 27.18) | -3.44 (-22.22, 15.19) |
| **AVAL** | -18.79 (-45.45, 7.75) | -2.73 (-27.18, 21.60) |  | -6.17 (-37.12, 24.48) |
| **CM** | -12.63 (-34.10, 9.02) | 3.44 (-15.19, 22.22) | 6.17 (-24.48, 37.12) |  |
| **24/26 weeks**  **Mean differences (95% credible interval)** | | | | |
| **Outcome: Force vital capacity (FVC)** | | | | |
|  | **PBO** | **ALG** | **AVAL** | **CM** |
| **PBO** |  | 3.80 (1.71, 5.91) | 6.44 (3.38, 9.49) | 6.68 (1.52, 11.81) |
| **ALG** | -3.80 (-5.91, -1.71) |  | 2.63 (0.42, 4.85) | 2.87 (-1.84, 7.58) |
| **AVAL** | -6.44 (-9.49, -3.38) | -2.63 (-4.85, -0.42) |  | 0.24 (-4.94, 5.45) |
| **CM** | -6.68 (-11.81, -1.52) | -2.87 (-7.58, 1.84) | -0.24 (-5.45, 4.94) |  |
| **Outcome: Six-minute walking distance (6MWD)** | | | | |
|  | **PBO** | **ALG** | **AVAL** | **CM** |
| **PBO** |  | 23.73 (8.91, 38.61) | 40.64 (9.40, 71.87) | 25.17 (-1.41, 52.00) |
| **ALG** | -23.73 (-38.61, -8.91) |  | 16.91 (-10.87, 44.72) | 1.44 (-20.83, 23.86) |
| **AVAL** | -40.64 (-71.87, -9.40) | -16.91 (-44.72, 10.87) |  | -15.46 (-50.73, 20.08) |
| **CM** | -25.17 (-52.00, 1.410 | -1.44 (-23.86, 20.23) | 15.46 (-20.08, 50.73) |  |

| **37/38 weeks**  **Mean differences (95% credible interval)** | | | | |
| --- | --- | --- | --- | --- |
| **Outcome: Force vital capacity (FVC)** | | | | |
|  | **PBO** | **ALG** | **AVAL** | **CM** |
| **PBO** |  | 3.60 (1.42, 5.80) | 5.25 (1.71, 8.83) | 0.11 (-7.13, 7.35) |
| **ALG** | -3.60 (-5.80, -1.42) |  | 1.65 (-1.14, 4.46) | -3.50 (-10.42, 3.43) |
| **AVAL** | -5.25 (-8.83, -1.71) | -1.65 (-4.46, 1.14) |  | -5.15 (-12.61, 2.32) |
| **CM** | -0.11 (-7.35, 7.13) | 3.50 (-3.43, 10.42) | 5.15 (-2.32, 12.61) |  |
| **Outcome: Six-minute walking distance (6MWD)** | | | | |
|  | **PBO** | **ALG** | **AVAL** | **CM** |
| **PBO** |  | 28.55 (9.85, 47.11) | 41.00 (10.14, 71.99) | 14.83 (-15.95, 45.58) |
| **ALG** | -28.55 (-47.11, -9.85) |  | 12.45 (-12.39, 37.65) | -13.72 (-38.40, 10.88) |
| **AVAL** | -41.00 (-71.99, 10.14) | -12.45 (37.65. 12.39) |  | -26.17 (-61.61, 9.08) |
| **CM** | -14.83 (-45.58, 15.95) | 13.72 (-10.88, 38.40) | 26.17 (9.08, 61.61) |  |
| **Last follow up**  **Mean differences (95% credible interval)** | | | | |
| **Outcome: Force vital capacity (FVC)** | | | | |
|  | **PBO** | **ALG** | **AVAL** | **CM** |
| **PBO** |  | 3.39 (1.03, 5.77) | 5.82 (2.35, 9.33) | 2.89 (-2.85, 8.70) |
| **ALG** | -3.39 (-5.77, -1.03) |  | 2.43 (-0.12, 4.99) | -0.51 (-5.78, 4.77) |
| **AVAL** | -5.82 (-9.33, -2.35) | -2.43 (-4.99, 0.12) |  | -2.93 (-8.75, 2.96) |
| **CM** | -2.89 (-8.70, 2.85) | 0.51 (-4.77, 5.78) | 2.93 (-2.96, 8.75) |  |
| **Outcome: Six-minute walking distance (6MWD)** | | | | |
|  | **PBO** | **ALG** | **AVAL** | **CM** |
| **PBO** |  | 26.27 (0.85, 51.70) | 55.09 (17.44, 92.78) | 20.48 (-24.27, 65.57) |
| **ALG** | -26.27 (-51.70, -0.85) |  | 28.82 (0.48, 57.23) | -5.79 (-43.35, 32.11) |
| **AVAL** | -55.09 (-92.78, -17.44) | -28.82 (-57.23, -0.48) |  | -34.61 (-81.60, 12.43) |
| **CM** | -20.48 (-65.57, 24.27) | 5.79 (-32.11, 43.35) | 34.61 (-12.43, 81.60) |  |
| **Notes:** Column treatment versus row treatment  **Abbreviation**: ALG, Alphaglucosidase; AVAL, Avalglucosidase; CM, Cipaglucosidase + Miglustat; PBO, placebo; FVC, forced vital capacity, 6MWD six-minute walking distance; | | | | |

**Table 10: Results of key review outcomes for the RCTs**

| **Outcome** | **LOTS(1)** | | | **COMET(2), (4)** | | | **PROPEL(3)** | | |
| --- | --- | --- | --- | --- | --- | --- | --- | --- | --- |
|  | Change from baseline at  week 78 (SD) | | Difference in means (95% CI) | Change from baseline at  week 49 (SE) | | Difference in means (95% CI) | Change from baseline at  week 52 (SE) | | Difference in means (95% CI) |
|  | ALG | PLB |  | AVAL | ALG |  | CM; N | ALG+PLB; N |  |
| **Motor function** | | | | | | | | | |
| 6MWD (m) | 25.13 (7.68) ^¶^ | -2.99 (10.80) ^¶^ | 28.12 (2.07, 54.17) | 32.21 (9.93) | 2.19 (10.40) | 30.01 (1.33, 58.69) | 20.8 (4.6); 85 | 7.2 (6.6); 37 | 13.7 (-1.2, 28.5) |
| 6MWD, % predicted | NR | NR | NR | 5.02 (1.54) | 0.31 (1.62) | 4.71 (0.25, 9.17) | 4.1 (0.8); 85 | 1.6 (1.0); 37 | 2.4 (-0.3, 5.0) |
| **Pulmonary function** | | | | | | | | | |
| FVC % predicted | 1.20 (0.70) ^¶^ | -2.20 (0.98) ^¶^ | 3.40 (1.03, 5.77) | 2.89 (0.88) | 0.46 (0.93) | 2.43 (-0.13, 4.99) | -0.9 (0.7); 84 | -4.0 (0.8); 37 | 2.7 (0.4, 5.0) |
| MIP % predicted | 3.48 (1.31) ^¶^ | -0.35 (1.84) ^¶^ | 3.83 (-0.60, 8.26) | 8.70 (2.09) | 4.29 (2.19) | 4.4 (-1.63, 10.44) | 2.1 (2.1); 84 | -2.7 (2.8); 37 | 4.2 (-3.4, 11.8) |
| **Muscular function** | | | | | | | | | |
| HHD lower | NR | NR | NR | 260.69 (46.07) | 153.72 (48.54) | 106.97 (-26.56, 240.5) | NR | NR | NR |
| HHD upper | NR | NR | NR | 173.54 (38.04) | 109.67 (38.98) | 63.87 (-44.76, 172.51) | NR | NR | NR |
| **Quality of life measures** | | | | | | | | | |
| EQ-5D 5L VAS | NR | NR | NR | 7.49 (1.99) | 2.20 (2.14) | 5.29 (-0.55, 11.2) | 0.03 (SE 1.54); 84 | 3.61 (SE 2.40); 36 | -3.58 (-4.29, -2.87) ^β^ |
| EQ-VAS score |  |  |  | 61.18 (SD = 15.90) | 66.69 (SD = 18.28) | Total (n = 99) is 63.88 (SD = 17.24) |  |  |  |

^¶^  SD calculated using the reported 95% CI

^β^ difference in means and 95% CI calculated (not available to extract)

ALG Alglucosidase alfa; AVAL Avalglucosidase alfa; CI confidence interval; CM is Cipaglucosidase + miglustat; EQ-5D VAS European Quality of Life 5 dimensions visual analogue scale; FVC forced vital capacity; HHD hand-held dynamometry; N sample size; NR means not reported; PLB is placebo; 6MWT Six-minute walk time; m metres; MIP maximal inspiratory pressure.

**Table 11: ERT subgroup results in the PROPEL trial**

| Outcome | **ERT-experienced** | | | | | **ERT-naive** | | | | |
| --- | --- | --- | --- | --- | --- | --- | --- | --- | --- | --- |
|  | **CM** | | **ALG+PLB** | | Mean difference (95% CI) | **CM** | | **ALG+PLB** | | Mean difference (95% CI) |
|  | Baseline mean | CFBL week 52 | Baseline mean | CFBL week 52 |  | Baseline mean | CFBL week 52 | Baseline mean | CFBL week 52 |  |
| 6MWD, m | 346·9 (110·2), n=65 | 16·9 (40·4) n=65 | 334·6 (114·0) n=30 | 0·0 (39·3) n=30 | LS 16·8 (0·2, 33·3) | 393·6 (112·4)  n=20 | 33·4 (48·7)  n=20 | 420·9 (135·7)  n=7 | 38·3 (29·3)  n=7 | LS −9·0 (−46·5, 35·0)  -4.9 (SE = 19.7) |
| FVC %  predicted | 67·9 (19·1)  n=65 | 0·1 (5·8)  n=64 | 67·5 (21·0)  n=30 | −4·0 (5·0)  n=30 | LS 3·5 (1·0, 6·0) | 80·2 (18·7)  n=20 | −4·1 (6·5)  n=20 | 79·1 (22·6)  n=7 | −3·6 (4·7)  n=7 | LS −2·0 (−8·9, 5·0)  -0.5 (SE 2.7) |
| MIP % predicted | 61·3 (27·9)  n=65 | 1·0 (20·1)  n=64 | 55·0 (16·9)  n=30 | −1·7 (8·1)  n=30 | LS 1·7 (−6·4, 9·7) | 63·5 (20·2)  n=20 | 5·6 (16·1)  n=20 | 80·7 (25·2)  n=7 | −6·9 (37·0)  n=7 | LS 5·3 (−22·6, 33·1) |

Numbers in brackets are standard deviations except where stated, LS Least squares

| **Outcome** | **LOTS n/N (%)** | | **COMET n/N (%)** | | **PROPEL n/N (%)** | |
| --- | --- | --- | --- | --- | --- | --- |
|  | **ALG** | **PLB** | **AVAL** | **ALG** | **C+M** | **ALG+PLB** |
| TEAEs potentially related to treatment | 32/60 (53) | 17/30 (57) | 23/51 (45) | 24/49 (49) | 26/85 (31) | 14/38 (37) |
| IAR | 17/60 (28) | 7/30 (23) | 13/51 (26) | 16/49 (33) | 21/85 (25) | 10/38 (26) |
| STEAEs | 13/60 (22) | 6/30 (20) | 8/51 (16) | 12/49 (25) | 8/85 (9) | 1/38 (3) |
| Discontinuations due to AEs | 2/60 (3) | 1/30 (3) | 0/51 (0) | 4/49 (8) | 3/85 (4) | 1/38 (3) |

**Table 12: Adverse Events in RCTs**

AE adverse events; ALG Alglucosidase; AVAL Avalglucosidase; C+M Cipaglucosidase + miglustat; IAR infusion associated reaction; PLB placebo; PR patient reported; STEAE serious treatment emergent adverse events; TEAE treatment emergent adverse events

**Table 13: Baseline characteristics of RCT extension cohorts**

| **Study** | **Treatment (N)** | **Mean age (SD)** | **% Male** | **Ht** | **Wt** | **Mean age at diagnosis (SD)** | **% Use of walking aid** | **Mean 6MWT, m (SD)** | **Mean 6MWT %**  **Predicted (SD)** | **Mean FVC**  **% predicted (SD)** |
| --- | --- | --- | --- | --- | --- | --- | --- | --- | --- | --- |
| **LOTS** | ALG-ALG (55) | NR | NR | NR | NR | NR | NR | NR | NR | NR |
|  | PLB-ALG (26) | NR | NR | NR | NR | NR | NR | NR | NR | NR |
|  | Total (81) | NR | NR | NR | NR | NR | NR | NR | NR | NR |
| **COMET** | AVAL-AVAL (51) | 47.0 (14.5) | 52.9 | NR | NR | NR | NR | 433.4 (111.8) | 62.6 (15.4) | 65.3 (17.1) |
|  | ALG-AVAL (44) | 50.7 (13.9) | 54.5 | NR | NR | NR | NR | 384.7 (139.6) | 55.8 (19.1) | 61.5 (13.5) |
|  | Total (96^*^) | 48.3 (14.7) | 53.1 | NR | NR | NR | NR | 410.8 (127.2) | 59.4 (17.5) | 63.6 (15.6) |
| **PROPEL^^^** | CM-CM (81) | 48.9 (13.5) | 40.7 | 171.2  (9.7) | 73.3  (15.3) | 40.3 (13.8) | NR | NR | NR | NR |
|  | ALG-CM (37) | 46.0 (13.5) | 51.4 | 171.2  (11.3) | 78.9  (26.8) | 37.2 (15.4) | NR | NR | NR | NR |
|  | Total (118) | 48.0 (13.5) | 44.1 | 171.2  (10.2) | 75.1  (19.7) | 39.3 (14.4) | NR | NR | NR | NR |

^*^ A new patient entered the study during the extension period, ^^^Baseline outcome data only reported for subgroups based on previous ERT status

ALG Alglucosidase alfa; AVAL Avalglucosidase alfa; CM Cipaglucosidase + Miglustat; FVC forced vital capacity; Ht height; N Sample size; PLB placebo; SD standard deviation; Wt weight; 6MWT six-minute walk time; m metres; % is percentage; In the extension period all patients crossed over to the intervention therapy.

**Table 14: Results of RCT extension studies**

| **Outcomes** | **LOTS Extension(7)** | | **COMET Extension(8, 9)** | | | | **PROPEL Extension(10)** | |
| --- | --- | --- | --- | --- | --- | --- | --- | --- |
|  | **CFBL at week 104** | **CFBL at week 130 (U.S. patients only)** | **CFBL at week 97(8, 11)** | | **CFBL at week 145(9, 12)** | | **CFBL week 104** | |
|  | **ALG-ALG**  **Mean (SD); N** | | **AVAL-AVAL**  **LS Mean (SE); N** | **ALG-AVAL**  **LS Mean (SE); N** | **AVAL-AVAL**  **LS Mean (SE); N** | **ALG-AVAL**  **LS Mean (SE); N** | **CM-CM**  **Mean (SD); N** | **ALG-CM**  **Mean (SD); N** |
| Motor function | | | | | | | | |
| 6MWT (m) | 21.3 (78); 53 | 22.9 (50.0); 27 | 18.60 (12.01); 42 | 4.56 (12.44); 41 | 20.65 (9.60) | 0.29 (10.42) | 381.54 (168.16); 74^¶, β^ | 359.94 (177.81); 33 ^¶, β^ |
| 6MWT, % predicted | NR | NR | 3.27 (6.44); 42  (1.48, 5.22) ^æ^ | 0.83 (6.14); 41  (-0.98, 2.71) ^æ^ | NR | NR | 4.44 (11.81); 74 ^¶^ | 1.49 (14.06); 33 ^¶^ |
| Pulmonary function | | | | | | | | |
| FVC % predicted | 0.8 (6.7); 53 | 0.2 (6.9); 27 | 2.65 (1.05); 43 | 0.36 (1.12); 35 | 1.43 (1.23) | 1.26 (1.35) | -1.66 (9.92); 71^¶^ | -3.64 (9.13); 31 ^¶^ |
| MIP, % predicted | 5.1 (10.7); 53 | NR | 9.99 (5.72); 40  (8.09, 11.76) ^æ^ | 5.29 (5.68); 33  (3.31, 7.23) ^æ^ | NR | NR | NR | NR |
| Quality of life | | | | | | | | |
| EQ-5D VAS | NR | NR | 72.02 (16.16) | 67.88 (19.61) | NR | NR | NR | NR |

^β^ mean at week 104 not CFBL, ^¶^ calculated using the values reported in the articles and in the subgroup results table, ^æ^ calculated by digitizing the published plot,

ALG Alglucosidase alfa; AVAL Avalglucosidase alfa; CFBL Change from baseline at randomisation, CM Cipaglucosidase + Miglustat; CFBL change from baseline; 6MWT six-minute walk time; m metres; ERT enzyme replacement therapy; FVC forced vital capacity; N group size; SD standard deviation.

**Table 14b: PROPEL extension study subgroup results**

| **Outcomes** | **CM-CM** | | **ALG-CM** | |
| --- | --- | --- | --- | --- |
|  | **ERT Experienced** | **ERT naive** | **ERT Experienced** | **ERT naive** |
|  | CFBL at 104 weeks  Mean (SD); N | CFBL at 104 weeks  Mean (SD); N | CFBL at 104 weeks  Mean (SD); N | CFBL at 104 weeks  Mean (SD); N |
| **Motor function** | | | | |
| 6MWT (m) | 14.2 (53.4); 56 | 38.8 (51.0); 18 | -8.8 (46.2); 26 | 48.3 (70.6); 7 |
| 6MWT (% predicted) | 3.1 (8.1); 56 | 8.6 (8.6); 18 | -0.5 (7.8); 26 | 8.9 (11.7); 7 |
| **Pulmonary function** | | | | |
| FVC % predicted | -0.6 (7.5); 53 | -4.8 (6.5); 17 | -3.8 (6.2); 24 | -3.1 (6.7); 5 |
| **Quality of life** | | | | |
| EQ-5D VAS | NR | NR | NR | NR |

ALG is Alglucosidase alfa; AVAL is Avalglucosidase alfa; CM is Cipaglucosidase + Miglustat; CFBL is changes from baseline; 6MWT is six-minute walk time; m is metres; % is percentage; ERT is enzyme replacement therapy; FVC is forced vital capacity; N is total number; SD is standard deviation; In the extension period all patients crossed over to the intervention therapy.

**Table 15: Extension study adverse events**

| **Outcome** | **LOTS** | **COMET** | | | | **PROPEL** |  |
| --- | --- | --- | --- | --- | --- | --- | --- |
|  |  | **97 Weeks** | | **145 Weeks** | | **104 weeks** |  |
|  | **ALG; n/N (%)** | **AVAL; n/N (%)** | **ALG; n/N (%)** | **AVAL; n (%)** | **ALG; n (%)** | **CM; n/N (%)** | **ALG; n/N (%)** |
| Any TEAEs | NR | 50/51 (98%) | 49*/44 (96.1%) | 50 (98%) ^a^ | 43/44 (97.7%) ^a^ |  | 36/37 (97.3) |
| TEAEs | 35% | 29/51 (56%) | 25/44 (56.8%) |  |  |  | 15/37 (40.5) |
| IAR | NR | 20/51 (39.2) | 21/44 (47.7) |  |  |  | 10 /37 (27.0) |
| Withdrawal due to AE and IAR | NR | 2/51 (3.9) | 3/44 (6.8) | 4 |  |  | 2/37 (5.4) |
| STEAEs | NR | 4/51 (7.8) | 2/44 (4.5) | 18 (35.3%) ^a^  (4 related to treatment) | 12 (27.3) ^a^  (2 were treatment related) |  | 2/37 (5.4) |
| Discontinuation | NR | 9/95 (5 is related to AE and 4 in non-treatment related) | | 5 (4 were treatment related) ^a^ | | 7/81 | 4/37 |

* Indicates that there is an error in the reporting of the number of patients with any TEAE in the article.

^a^ indicates values were extracted from abstract not full text.

AE is adverse events; ALG is Alglucosidase alfa; AVAL is Avalglucosidase alfa; CM is Cipaglucosidase + Miglustat; IAR is infusion associated reaction; PLB is placebo; STEAE is serious treatment emergent adverse events; TEAE is treatment emergent adverse events; In the extension period all patients crossed over to the intervention therapy.

**Table 16: Relative treatment effects of FVC and 6MWD (RE and FE model) of all ERT versus placebo at time varying timepoints**

| **Mean differences (95% credible interval); FE model** | | | |
| --- | --- | --- | --- |
|  | Treatments comparison | FVC % predicted | 6MWD |
| 12/13 weeks | ERT vs PBO | 2.21 (1.22, 3.21) | 18.19 (11.47, 31.47) |
| 25/26 weeks | ERT vs PBO | 1.38 (0.40, 2.37) | 22.91 (14.48, 31.47) |
| 37/38 weeks | ERT vs PBO | 2.74 (1.54, 3.95) | 28.29 (18.76, 37.79) |
| 49/52 weeks | ERT vs PBO | 2.40 (1.20, 3.61) | 24.62 (13.9, 35.39) |
| LFU | ERT vs PBO | 2.91 (1.67, 4.16) | 28.50 (16.08, 40.90) |
| **Mean differences (95% credible interval); RE model** | | | |
|  | Treatments comparison | FVC % predicted | 6MWD |
| 12/13 weeks | ERT vs PBO | 2.04 (-0.01, 3.83) | 18.28 (10.99, 25.28) |
| 25/26 weeks | ERT vs PBO | 0.63 (-2.45, 3.35) | 22.83 (13.94, 31.66) |
| 37/38 weeks | ERT vs PBO | 2.48 (-0.58, 5.42) | 28.23 (18.45, 38.05) |
| 49/52 weeks | ERT vs PBO | 1.69 (-1.57, 4.69) | 24.66 (13.76, 35.61) |
| LFU | ERT vs PBO | 2.27 (-0.97, 5.23) | 28.52 (15.92, 41.02) |
| **Abbreviation**: ERT, enzyme replacement therapy; PBO, placebo; FVC, forced vital capacity, RE, random effects, FE, fixed effect; 6MWD six-minute walking distance; LFU is last follow up time | | | |

**References**

1. van der Ploeg AT, Clemens PR, Corzo D, Escolar DM, Florence J, Groeneveld GJ, et al. A randomized study of alglucosidase alfa in late-onset Pompe's disease. N Engl J Med. [Multicenter Study Randomized Controlled Trial Research Support, Non-U.S. Gov't]. 2010 Apr 15;362(15):1396-406.

2. Diaz-Manera J, Kishnani PS, Kushlaf H, Ladha S, Mozaffar T, Straub V, et al. Safety and efficacy of avalglucosidase alfa versus alglucosidase alfa in patients with late-onset Pompe disease (COMET): a phase 3, randomised, multicentre trial. Lancet Neurology. [Multicenter Study Randomized Controlled Trial Research Support, Non-U.S. Gov't]. 2021 12;20(12):1012-26.

3. Schoser B, Roberts M, Byrne BJ, Sitaraman S, Jiang H, Laforet P, et al. Safety and efficacy of cipaglucosidase alfa plus miglustat versus alglucosidase alfa plus placebo in late-onset Pompe disease (PROPEL): an international, randomised, double-blind, parallel-group, phase 3 trial. Lancet Neurology. [Clinical Trial, Phase III Randomized Controlled Trial Research Support, Non-U.S. Gov't]. 2021 12;20(12):1027-37.

4. Toscano A, Pollissard L, Msihid J, van der Beek N, Kishnani PS, Dimachkie MM, et al. Effect of avalglucosidase alfa on disease-specific and general patient-reported outcomes in treatment-naive adults with late-onset Pompe disease compared with alglucosidase alfa: Meaningful change analyses from the Phase 3 COMET trial. Molecular Genetics & Metabolism. 2024;141:108121.

5. Plummer M, Stukalov A, Denwood M. Package ‘rjags’: Bayesian graphical models using MCMC. CRAN; 2024 [cited 2025 14 February]; Available from: <https://cran.r-project.org/web/packages/rjags/rjags.pdf>.

6. Dias S, Welton NJ, Sutton AJ, Ades AE. NICE DSU Technical Support Document 2: A generalised linear modelling framework for pairwise and network meta-analysis of randomised controlled trials. Sheffield: Decision Support Unit, ScHARR, University of Sheffield 2014.

7. van der Ploeg AT, Barohn R, Carlson L, Charrow J, Clemens PR, Hopkin RJ, et al. Open-label extension study following the Late-Onset Treatment Study (LOTS) of alglucosidase alfa. Molecular Genetics & Metabolism. [Multicenter Study Randomized Controlled Trial Research Support, Non-U.S. Gov't]. 2012 Nov;107(3):456-61.

8. Kishnani PS, Diaz-Manera J, Toscano A, Clemens PR, Ladha S, Berger KI, et al. Efficacy and Safety of Avalglucosidase Alfa in Patients With Late-Onset Pompe Disease After 97 Weeks: A Phase 3 Randomized Clinical Trial. JAMA Neurology. [Randomized Controlled Trial Clinical Trial, Phase III Research Support, Non-U.S. Gov't]. 2023 06 01;80(6):558-67.

9. Kishnani PS, Diaz-Manera J, Kushlaf H, Ladha S, Mozaffar T, Straub V, et al. Efficacy and safety of avalglucosidase alfa in participants with late-onset Pompe disease after 145 weeks of treatment during the COMET trial. Mol Genet Metab. [Journal article; Conference proceeding]. 2023;138(2).

10. Schoser B, Kishnani PS, Bratkovic D, Byrne BJ, Claeys KG, Diaz-Manera J, et al. 104-week efficacy and safety of cipaglucosidase alfa plus miglustat in adults with late-onset Pompe disease: a phase III open-label extension study (ATB200-07). J Neurol. 2024;271:2810-23.

11. Henderson R, Kishnani PS, Diaz-Manera J, Kushlaf H, Ladha S, Mozaffar T, et al. Efficacy and Safety of Avalglucosidase Alfa in Participants with Late-Onset Pompe Disease after 97 Weeks of Treatment during the Comet Trial. BMJ Neurology Open. 2022;4(Supplement 1):A30-A1.

12. Henderson RD, Schoser B, Kishnani PS, Kushlaf H, Ladha S, Mozaffar T, et al. EFFICACY and SAFETY of AVALGLUCOSIDASE ALFA in PARTICIPANTS with LATE-ONSET POMPE DISEASE after 145 WEEKS' TREATMENT: PHASE 3 COMET TRIAL. BMJ Neurology Open. 2023;5(suppl 1):A17.

13. European Medicines Agency. CHMP Assessment report Pombiliti. Amsterdam: EMA2022.

14. European Medicines Evaluation Agency. CHMP Assessment report for myozyme. Amsterdam: EMEA2009.
