# Supplementary material for: Enzyme replacement therapy for the treatment of late onset Pompe disease: A systematic review and network meta-analysis
Source: Orphanet J Rare Dis. 2025 Aug 21;20:451. doi: 10.1186/s13023-025-03981-0 (PMC12372379; doi:10.1186/s13023-025-03981-0)
Supplement: Supplementary file 3 — Supplementary Material 3 [file 13023_2025_3981_MOESM3_ESM.docx]

**Additional File 3 – ERT registry studies and other prospective ERT studies**

**Table 1 Baseline characteristics of ERT registry studies**

| Study, N, ERT | Age at ERT start (years) | Age at onset  years | % Male | % using wheelchair | % respiratory support | 6MWT, m | 6MWT %  Predicted | FVC  % predicted |
| --- | --- | --- | --- | --- | --- | --- | --- | --- |
| Pompe Registry (Sanofi, Genzyme) | | | | | | | | |
| Berger et al 2024,(1) 485, ALG | 44.9^*^ | 34.3^*^ | 51 | 7 | 28 Non-invasive  0 invasive | 380^*^  N=286 | NR | 67.1^*^ |
| Stockton et al 2020(2) 396, ALG | 45^*^ | 33.7^*^ | 50 | 13 (n=187) | 16 (n=188) | NR | NR | 66.9^*^ |
| Marcondes et al,(3) 38 (LA), 1390 (RoW), ALG, CA | NR | LA 26.3  RoW 30.0 | LA 47  RoW 50 | NR | RoW 8% invasive | NR | NR | RoW 67% had an FVC <80% |
| French Pompe Registry | | | | | | | | |
| Semplicini et al,(4) 158, ALG | 50.9 | 37 | 48 | 18 | 52 | NR | 57.0 | 64.4 |
| Lefeuvre 2023, 177 on ERT, ALG(5) | 49.6^*^ | NR | 49 | NR | NR | 431 | NR | NR |
| Tard et al 2024, ALG - AVAL (switching study), 29(6) | 45.3  56.2 at switch | NR | NR | NR | NR | 213 (147) at switch | NR | NR |
| Spanish Pompe Registry | | | | | | | | |
| Martinez – Marin et al (2024)(7), 81 on ERT | 29.1 | NR | NR | NR | NR | NR | NR | NR |

^*^ Median, ALG Alglucosidase alfa, CA conference abstract, FVC Forced vital capacity, LA Latin America, 6MWT six-minute walk test, RoW Rest of the world; NR not reported.

**Table 2 Results of ERT registry studies**

| Study, N | 6MWT (m), N (if different from total) | 6MWT %  predicted | FVC % predicted, N (if different from total) | Muscle function outcomes | Other outcomes |
| --- | --- | --- | --- | --- | --- |
| **Pompe Registry (Sanofi, Genzyme)** | | | | | |
| Berger et al 2024,(1) 485, ALG | NR | NR | Median FU 8.3 years  1.83 (0.66, 3.01) increase /year for first 6 months  -0.54 (−0.79, −0.30) decrease /year from 6 months to 5 years  -1.0 (−1.36, −0.63) decrease /year from 5-13 years | NR | 34 patients (7%) died during follow up at a median age of 60.3 years. |
| Stockton et al 2020(2)396, ALG | NR | NR | Median FU 4 years  change in FVC over time:  -0.17 p/y | NR | 26 patients (16.5%) began non-invasive respiratory support during the 5-year FU. This was nighttime only for 22 patients. |
| Marcondes et al, (3) 38 (LA)  1390 (RoW), ALG, CA | RoW: At 2 years 8.7% had a >20% decline | NR | RoW: At 2 years 22.7% had a >10% decline | NR | NR |
| **French Pompe Registry** | | | | | |
| Semplicini et al,(4) 158, ALG | NR | Median FU 5.3 years 1.4% increase p/y up to 2.2 years, followed by 2.3% decline | Median FU 5.3 years  −0.9 decrease /year | MFM D2 sub-score:  −1.0% /year, p< .001) D3 −0.2%/ year, p < .05), | 15 patients (9%) died at mean age 65.2 years and mean ERT duration 4.3 years |
| Lefeuvre 2023, 177 treated with ERT, ALG(5) | 6MWD:  Baseline: 431  10-year FU: slope = 0.85, “mean progression of -1.00”; significant decrease over time (p<0.001) | NR | Sitting FVC: mean change ‘over time’ 0.02  Supine FVC: mean change ‘over time’ 0.01 | NR | Median time until walking ability progression (use of a walking stick, walker or wheelchair) = 9.4 years and time until respiratory function progression (use of any ventilation) = 14.8 years. 31 patients died at a mean age of around 66 years. |
| Tard et al 2024, ALG – ALG to AVAL (switching study), 29(6) | Decrease of 63m pre-switch vs a decrease of 1 m one year post-switch. | NR | No statistically significant difference at 1 year when compared with pre-switch data. | NR | NR |
| **Spanish Pompe Registry** | | | | | |
| Martinez – Marin et al (2024)(7), 81 treated with ERT | Estimated yearly decline (n=58):  < 5 years: -5.16 (-8.3 to -2.03)  5–10 years: -8.59 (-15.45 to -1.72)  >10 years: -8.74 (-237.2 to 219.7) | NR | Estimated yearly decline (n=69):  < 5 years: 0.46 (-1.48 to 0.57)  5–10 years: -1.03 (-1.75 to 3.81)  >10 years: -1.24 (-1.2 to 2.15) | NR | NR |

* Median, CA Conference abstract, FU Follow up, p/y per year, LA Latin America, MFM Motor Function Measurement, RoW Rest of the world

**Table 3: Registry studies reporting adverse event data**

| **Authors** | **Adverse events** | | | | | |
| --- | --- | --- | --- | --- | --- | --- |
|  | TEAE  n/N (%) | TEAE related to treatment n/N (%) | IAR  n/N (%) | Withdrawal due to TEAE n/N (%) | STEAE  n/N (%) | Discontinuation  N (%) |
| Semplicini et al, (4) 158, ALG | NR | NR | 14/158 (9) | NR | NR | 26 (17.3)  Median FU 5.3 years |

**Table 4: Baseline characteristics of other prospective ERT studies**

| Study, N | Country | Age at ERT start years | Age at onset of disease | % Male | % using wheelchair | % respiratory support | 6MWT, m (SD) | 6MWT %  Predicted (SD) | Sitting FVC  % predicted (SD) |
| --- | --- | --- | --- | --- | --- | --- | --- | --- | --- |
| Alglucosidase Alfa studies | | | | | | | | | |
| Anderson et al (2014),(8) 62 | UK | 45.5 | NR | 60 | 16 | 39 | 246 | NR | 59.6 |
| Angelini et al (2012),(9) 74 | Italy | 43 | 28.3 | 45 | 10 | 36 | 320 (161) (n=58) | NR | 65.2 (26.5) (n=69) |
| Angelini et al (2012),(10) 40 (subgroup of (9)) | Italy | 51 | NR | 45 | 0 | 35 | 319 (n=32) | NR | NR |
| Bembi et al (2010),(11) 24 | Italy (Juvenile, 7) | 12 | 2.5 | 71 | 4 | 29 | 572.9^*^ | NR | 83 (45) |
|  | Italy (Adult, 17) | 47.6 | 26.6 | 53 |  | 65 | 116.6^*^ | NR | 52 (26) |
| Claeys et al (2022),(12) 12 | Belgium | All on ERT before the study: mean of 8.8 yrs | 32.8 | 42 | 0 | 33 | 451.9 (143.3) | NR | 82.8 (24.89) |
| Clemens et al (2017) (13); 68, CA | Same setting as LOTS | 45.8 | 28.7 | 53 | 50 | 38 | NR | NR | Estimated annual decline 1.3% per year |
| De Vries et al (2012),(14) 69 | Netherlands | 52.1^*^ | 30.8^*^ | 52 | 40 | 37 | NR | NR | 68.3^*^ |
| Ditters et al (2023),(15); 121  Ditters et al (2023),(16)100 | Netherlands | 52.2^*^ | 33.5^*^ | 49 | NR | NR | NR | NR | NR |
|  | Netherlands | 50.1^*^ adult  10.5^*^ childhood onset | NR | 42 adult  60 childhood onset | NR | NR | NR | NR | NR |
| Gungor et al (2013),(17)204^æ^/283 | Netherlands, UK, US, Germany, Australia, and Canada | 51^*^ | NR | 49 | 18 | 14 | NR | NR | NR |
| Gungor et al (2016),(18); (19)174 | Netherlands, UK, US, Germany, Australia, and Canada | 50^*^ | NR | 47 | 52 | 48 | NR | NR | NR |
| Hahn et al (2018),(20) 26 | US | 6.0 | NR | 73 | NR | NR | NR | NR | NR |
| Harlaar et al (2019), 30 LOTS trial patients(21-24) | Netherlands and France | 49^*^ | NR | 47 | 23 | 23 | NR | 49^*^ | 54^*^ |
| Hartung et al (2007),(25)11, CA | Germany | NR | NR | NR | NR | NR | 336 (215) | NR | 2.35 (1.06) |
| Kuperus et al (2017),(26) 102 (88 on ERT) | Netherlands | 52^*^ | 33^*^ | 52 | 36 | 29 | 376^*^, 53 | NR | NR |
| Kuperus et al (2018)(27); 112 | Netherlands | 49* | 31* | 50 | 31 | 22 | 417 | NR | 57 |
| Ravaglia et al (2022)(28), 18 | Italy | 53^*^ | 36^*^ | 39 | 17 | 44 | 367 | 67 (23), n=12 | 73.8 (21.3), n=12 |
| Ravaglia et al (2012)(29), 16 | Italy | NR | 31 | 44 | 0 | 44 | 339 for non-responders n=7 | NR | 59 (27.5) |
| Regnery et al (2012)(30); 38 | Germany | 50.7 | 36.2 | 47 | 16 | 34 | 312 (165.5); n=21 | NR | 80.27 (14.1); n=28 |
| Strothotte et al (2010) (31); 44 | Germany | 48.9 | NR | 55 | 18.9 | 43.2 | 341 (149.5); n=22 | NR | 69.9 (28.1); n=33 |
| Van der Meijden et al (2018)(32); 319; 126 at risk of wheelchair, 125 at risk of respiratory support | Canada, Germany, Netherlands, US, France, and UK | 48* – WC  49* – RS | 39* (based on n=189 at risk of using wheelchair including those who started ERT and NH)  39* (based on n = 177 at risk of using RS including those who started ERT and NH) | 48* (ERT and non-ERT)  38* (ERT and non-ERT) | 0 | 0 | NR | NR | NR |
| Van der Meijden et al (2018)(33); 17 | Netherlands, Belgium, UK, USA, and Germany. | 11.9* | 2.5* (on text) and 3* (on the table). | 65 | 18 | 18 | NR | 79* | 87* |
| Van der Ploeg (2016) (34);  Thurberg et al (2015) (35); 16 | US, UK, Germany, and the Netherlands | 51.6 (13.69) | 40 (11.58) | 44 | 0% | 0% | 449.9 (208.0); n=15 | NR | Upright: 76.4 (15.63); n=15 |
| Van Kooten et al (2020)(36); 111 | Netherlands | 42.7* (G1)  56.6* (G2A)  66 * (G2B) | NR | 50 (G1)  100 (G2A)  50 (G2B) | 60 (G1)  90 (G2A)  75 (G2B) | 20 (G1)  90 (G2A)  50 (G2B) | NR | NR | NR |
| Avalglucosidase alfa studies | | | | | | | | | |
| NEO1 and NEO-EXT, Dimachkie et al (2022) (37), (38); (39); (40)24,  Schoser et al (2020) (41); (42); (43)  Mozaffar et al (2023)(44) | US, France, Germany, Belgium, Denmark, Netherlands, UK  Naïve, 10 | NR | 43.3 (23.79), 8 | 50 | 0 | 0 | 449 (118) | <45 years: 71.6 (9.9)  >/= 45 years: 56.2 (19.2) | 69.2 (19.3) |
|  | US, France, Germany, Belgium, Denmark, Netherlands, UK  Experienced (ALG), 14 | NR | 36.3 (16.39), 9 | 64 | 0 | 0 | 440 (141) | < 45 years: 70.3 (13.4)  >/= 45 years: 54.2 (18.5) | 77.3 (16.4) |
| Cipaglucosidase + Miglustat studies | | | | | | | | | |
| Byrne et al (2023)(45)29 | Australia, UK, US, Netherlands, New Zealand, and Germany:  Cohort 1, 11 | NR | NR | 82 | NR | NR | 397.2 (96.8) | 61 (13.4) | 52.6 (13.9) |
|  | Cohort 2, 6 | NR | NR | 67 | 100 | NR | NA | NA | 42.3 (28.2) |
|  | Cohort 4, 6 | NR | NR | 33 | NR | NR | 387.3 (161.3) | 59 (21.4) | 65.3 (21.1) |
|  | Experienced | NR |  |  | NR | NR |  | 60.2 (16.2), n=16 | 57.4 (17.4), n=16 |
|  | Naïve (Cohort 3) | NR | NR | 20 | NR | NR | 396 (75.2) | 67.8 (12.6), n=6 | 57.2 (20.8), n=6 |

Continuous data are means (standard deviation) unless otherwise indicated, ^*^ Medians, CA conference abstract, ^æ^ Indicate ERT patients out of the total number (N) in the study. Cohort 1 indicates 2 – 6 years ERT experienced patients; Cohort 2 indicates ≥ 2 years ERT experienced non-ambulatory patients; Cohort 3 indicates ERT naive patients; Cohort 4 indicates ≥ 7 years ERT experienced patients; NR not reported; N sample size; US United State of America; UK United Kingdom; G1 is group 1 which is made up of patients that discontinue ERT for personal reasons, G2A is group 2A which are deceased patients related to pompe disease and G2B is group 2B which are deceased patients non-related to pompe disease; WC wheelchair, RS respiratory support.

**Table 5: Results of other prospective ERT studies**

| Study, N | Subgroups | 6MWT (m) | 6MWT %  predicted | FVC % predicted | Muscle function or strength outcomes | Other outcomes |
| --- | --- | --- | --- | --- | --- | --- |
| Alglucosidase studies | | | | | | |
| Anderson et al (2014),(8) 62 (59 on ERT) |  | CFB with 95% CI, n=20:  <12 months: 43.7 (13.8, 73.6)  1-3 years: 51.3 (29.1, 73.5)  >3 years: 16.1 (−21.4, 53.6) | NR | CFB 95% CI, n=57:  <12 months: 1.77 (−0.75, 4.29)  1-3 years: −0.21 (−2.55, 2.14)  >3 years: −2.11 (−5.68, 1.46) | MRC, CFB 95% CI, n=53:  <12 months: 3.53 (1.39, 5.66)  1-3 years: 4.04 (2.26, 5.83)  >3 years: 1.30 (−1.92, 4.52) | Safety data – see table below |
| Angelini et al (2012),(9) 74 |  | Baseline: 320  End of follow up: 383, p<0.001, n=58  Follow ranged from 12 to 54 months. | NR | Baseline: 65.2  4 years: 66.5, p=0.22, n=69 | NR | 6 patients stopped ventilatory support and 2 started NIV. Of 21 patients continuing with NIV mean hours dropped from 15.6 to 12.1 hrs/day |
| Angelini et al (2012), 40(10)(subgroup of (9)) |  | Baseline: 319  1 Year: 371, p<0.001, n=32 | NR | NR | NR | NR |
| Bembi et al (2010),(11) 24 | Juvenile, 7 | Baseline: 572.9^*^  1 year: 589^*^, p<0.03  2 years: 630^*^, p<0.03  3 years: 664^*^, p=0.01 | NR | Baseline: 54^*^  1 year: 56^*^  2 years: 60^*^  3 years: 59.5^*^ not ss across timepoints | NR | Of the 13 patients needing ventilatory support at baseline, this dropped to 8 patients at the end of the study. The median daily ventilation for the remaining 8 patients reduced from 14 to 8 at year 1, 2, 3. |
|  | Adult, 17 | Baseline: 116.6^*^  1 year: 213.1^*^, p<0.001  2 years: 206^*^, p<0.001  3 years: 265^*^, p<0.001 | NR |  | NR |  |
| Claeys et al (2022),(12) 12 |  | CFB with 95% CI  6 months: -19.1 (-58.8 to 20.6)  1 year: -41.3 (-86.9 to 4.3)  18 months: -28.5 (-63.9 to 6.9)  2 years: -60.6 (-92.0 to -29.1) | NR | Sitting (n=12):  Baseline: 82.8 (24.89)  2 years: 80.3 (25.08)  Supine (n=11):  Baseline: 65.6 (21.97)  2 years: 65.10 (25.77)  Neither result was ss. | MRC:  Baseline: 67.2 (8.2)  6 months: 70.4 (7.7)  1 year: 71.1 (8.3)  18 months: 70.4 (8.3)  2 years: 71.3 (8.1)  Not ss for all timepoints | NR |
| Clemens et al (2017) ;(13) 68, CA |  | NR | Increased for first 2 – 3 years, then a modest decline over the next 3 years and a decrease of 6.4% over the first 6 years. | % predicted FVC declined by 0.78% (after having received up to 9 years of ERT). | NR | 71% of 42 patients who were ventilator free at baseline remained ventilator free. 53% of non-ambulatory at baseline remain non-ambulatory. |
| De Vries et al (2012),(14) 69 |  | NR | NR | Median FU 23 months:  Upright: increased by 0.1%/y, p=0.92, n=62 | Median FU 23 months :  MRC: 1.4%/y, p<0.001, n=69  HHD: 4%/y, p<0.001, n=64  QMFT: 0.7%/y, p=0.14, n=69 | No change in median  no. of hours of ventilation per day.  Also see safety data table |
| Ditters et al (2023),(15) 121; (16)100 |  | NR | NR | NR | NR | IAR (see safety data table) |
| Gungor et al (2013),(17) 204^æ^/283 |  | NR | NR | NR | NR | ERT positively associated with survival:  HR = 0.41 (0.19, 0.87) |
| Gungor et al (2016),(18); (19)174 |  | NR | NR | NR | NR | Change in points per year  SF-36 PCS, 0-2 years: 1.49 (0.76, 2.21)  >2 years: -0.15 (-0.43; 0.13)  SF-36 MCS, 0-2 years: 1.03 (-0.07, 2.13)  >2 years: 0.02 (-0.41, 0.46) |
| Hahn et al (2018),(20) 26 (22 patients were analysed) |  | NR | NR | Upright FVC decreased ≥ 15% predicted at 1 year: 11.1% (95% CI: 0.3% to 48.2%) | GMFM-88:  Baseline: 70.9%^*^  1 Year: 85.9%^*^  CFB: 0.2%^*^ | At 1 year:  Invasive ventilator free survival: 88.7% (61.4% - 97.1%). No deaths. |
| Harlaar et al (2019), 30 LOTS trial patients(21-24) |  | NR | 10 years FU (n = 7): -22.2%, p<0.001 | Upright at 10 years FU (n = 11): -10.98 %, p<0.001  Supine at 10 years FU (n = 8): -9.21%, p<0.001 | MRC scores were significantly lower after 10 years (graph only, split by sex) | At end of FU 13 (43%) were partially of fully wheelchair dependent compared to 7 at ERT start. 24 (80%) needed NIV compared to 7 (23%) at ERT start.  MIP: -1.79% at 10 years  MEP: -2.50% at 10 years |
| Hartung et al (2007),(25) 11, CA |  | Baseline: 336 (215)  6 months: 383 (220)  CFB: 47 Statistical significance NR | NR | Only FVC reported (not % predicted) | NR | NR |
| Kuperus et al (2017),(26) 88 | ERT | Baseline: 376^*^  5 years: 416^*^, n=53  CFB: 40.9, p=0.03 | NR | Upright:  5 years: CFB: -0.1, p=0.84, n=84  Supine:  5 years: CFB: -2.9, p=0.005, n=74 | CFBL at 5 years:  MRC score: 0.7, p=0.25, n=88  HHD score: 8.4, p<0.001, n=84  QFMT score: -0.2, p=0.87  R-Pact: 3.6, p=0.004 | 8 and 9 additional patients needed a ventilator or wheelchair respectively at study end.  MIP: CFB: -0.5%, p=0.81, n=83  MEP: CFB: 2.6%, p=0.18, n=83 |
| Kuperus et al (2018)(27) 112  *Values were digitised from plots* | II ACE genotype  N=32 | Baseline: 424  1 year: 433, 2 years: 434,  3 years: 427, 4 years: 417  5 years: 406 | NR | Baseline: 80  1 year: 80, 2 years: 80  3 years: 79, 4 years: 78  5 years: 78 | NR | NR |
|  | DD ACE genotype  N=41 | Baseline: 365  1 year: 368, 2 years: 371  3 years: 371, 4 years: 365  5 years: 354 | NR | Baseline: 66  1 year: 68, 2 years: 68  3 years: 67, 4 years: 66  5 years: 65 | NR |  |
|  | ID ACE genotype  N=58 | Baseline: 399  1 year: 428, 2 years: 441  3 years: 441, 4 years: 430  5 years: 415 | NR | Baseline: 64  1 year: 65, 2 years: 65  3 years: 65, 4 years: 66  5 years: 65 | NR |  |
| Ravaglia et al (2022),(28) 18 |  | Baseline: 367  1 year: 424, p=0.01, n=12  3 years: 377  CFB (*digitized from plot*):  6 years FU: -17 (107.98)  15 years FU: -58 (192.73) | NR | NR | NR | NR |
| Ravaglia et al (2012)(29); 16 |  | 24 months (*digitized from plot):*  Mean % change in 6MWT 38.35 (51.7) | NR | NR | NR | Muscle strength, endurance and mass improved in 14 (87%), 13 (81%) and 11 (69%) of patients respectively. |
| Regnery et al (2012)(30); 38 |  | All n=21, baseline: 312 (165.5)  1 year: 344 (p=0.006)  2 years: 356.4 (p=0.03)  3 years: 325.6 (p=0.49) | NR | All n=28, baseline: 80.27,  1 year: 79.19  2 years: 78.62  3 years: 77.19  All results were not ss. | MRC, all n=38:  Baseline: 42.29  1 year: 41.92  2 years: 43.89  3 years: 41.19  All results were not ss. | NR |
| Strothotte et al (2010)(31); 44 |  | Baseline: 342^*^(mean 341)  12 months: 412^*^(mean 393) ; 22  p<0.03 for comparison of means | NR | Baseline: 69.9 (SD: 28.1)  12 months: 70 (SD: 26.9); 33 | MRC: n=34  Baseline: 41.5  12 months: 42, p=0.31 | NR |
| Van der Meijden et al (2018) (32); 319  126 at risk of wheelchair, 125 at risk of respiratory support |  | NR | NR | NR | NR | 16 ERT patients started using wheelchair and 28 ERT patients started respiratory support during the follow up. The hazard ratios for WC and RS comparing patients treated with ERT and untreated patients are 0.36 (0.17, 0.75) and 1.23 (0.61, 2.47) respectively. |
| Van der Meijden et al (2018)(33); 17 |  | NR | 7 years: increased by 7.4% (95% CI: 2.4 - 12.3); 14 | Sitting position  Baseline: 87^*^ (16-104%)  7 years: -5.2^*^% (95% CI: 0.05-10.4)  Supine position  Baseline: 85^*^ (39-109%)  7 years: -4.7^*^% (95% CI: -4.5 - 13.9) | QMFT n=(15):  Baseline: 92^*^  7 years: increased by 9.2% (95% CI: 1.8 – 16.6)  HHD (14 patients):  Baseline: 57^*^  7 years: increased by 17.8% (95% CI: -3.4 - 39)  MRC (17 patients):  Baseline: 91.7^*^  7 years: decreased by 1.3% (95% CI: -0.7 - 3.28) | NR |
| Van der Ploeg (2016)(34); Thurberg et al (2015)(35); 16 |  | 6 months: 471.2  CFB: 37.3, p=0.02 | NR | Upright, 6 months: 77.6  CFB: 1.8, p=0.67  Supine: 6 months: 60.8  CFBL: 2.9, p=0.41 | HHD (n=15), 6 months  Upper body:  CFB: 43.2, p=0.55  Lower body:  CFB: 188.3, p=0.09 | PedsQoL Fatigue scale (n=15), 6 months, CFB: 8.1  MIP% (n=13 for upright; n=6 for supine):  Upright: CFB: 1.6  Supine: CFB: -8.2  %MEP (n=13 for upright; n=6 for supine):  Upright: CFB: 2.4  Supine: CFB: – 11.6  Other measures are GSGMC, GMFS, QMT |
| Van Kooten et al (2020)(36); 111 |  | NR | NR | NR | NR | Discontinuation reported in AE table below |
| Avalglucosidase alfa studies | | | | | | |
| NEO1 and NEO-EXT, Dimachkie et al (2022)(37, 38); (39); (40)24,  Schoser et al (2020); (42); (43); Mozaffar et al (2023)(44); Schoser et al (2020)(41); 24, CA | Naive | Note  Baseline: 449  CFB 12 weeks: -46.5, n=2 | Over up to 6 years: -0.70%/ year (95% CI: -1.57, 0.17) ^β^ | Over up to 6 years:  -0.47%/year (95% CI: -1.19, 0.24) ^β^ | NR | MIP% predicted of 1.51 per year (-1.04 to 1.34) and MEP% predicted of 0.73 per year (-0.49 to 1.95) |
|  | Experienced | Baseline: 440  CFB 12 weeks: -53.0, n=3 | -0.846%/ year (95% CI: -1.57, -0.13) ^β^ | Baseline: 77.3 (16.4)  -0.648%/year (95% CI: -1.06, -0.24) ^β^ | NR | MIP% predicted of -0.63 per year (-1.56 to 0.30) and MEP% predicted of 0.95 per year (-0.27 to 2.17) |
|  | Naïve  5.5 years | NR | -0.965 (-1.89, -0.04) | Slope: 0.396 (-0.35, 1.14) | NR | MIP%: 0.74 (-0.61, 2.10)  MEP%: 0.70 (-0.69, 2.09) |
|  | Switch  5.5 years | NR | -1.216 (-2.03, -0.41) | Slope: -0.331 (-0.78, 0.12) | NR | MIP%; -0.95 (-2.00, 0.11)  MEP%: 1.19 (-0.15, 2.53) |
| Cipaglucosidase + Miglustat studies | | | | | | |
| Byrne et al (2023)(45); 29 | ERT Experienced (excluding non-ambulatory) | CFB 4 years: 20.7, n=9 | 4 years: 66.2, 9  CFB 4 years: 5.9, n=9 | 4 years: 55.7, 6  CFB 4 years: 1.0, n=6 | MMT LES:  Baseline: 30; 15  4 years: 35; 8 | Additional outcomes in supplementary table s2 |
|  | ERT-Naïve | CFB 4 years: 52.2, n=4 | 4 years: 82.8, 4  CFB 4 years: 11.7, n=4 | 4 years: 37, 4  CFB 4 years: 8.3, n=4 | MMT LES:  Baseline: 29, 5  4 years: 30, 4 | NR |

Continuous data are means unless otherwise indicated; ^*^Indicates median; ^β^ Indicates slope; CA Conference abstract; CFB Change from baseline; ERT enzyme replacement therapy; FU Follow up; HHD handheld dynamometry; LES lower extremities score; MMT manual muscle test; MRC Medical Research Council sum score; NIV Non-invasive ventilation; QMFT Quick motor function test; ss statistically significant

**Table 6 Adverse events reported in other prospective ERT studies**

| **Authors** | Deaths | **Adverse events** | | | | | |
| --- | --- | --- | --- | --- | --- | --- | --- |
|  |  | TEAE  n/N (%) | TEAE related to treatment n/N (%) | IAR  n/N (%) | Withdrawal due to TEAE n/N (%) | SAE  n/N (%) | Discontinuation  n |
| Anderson et al (2014)(8) |  |  |  |  |  |  |  |
| Angelini et al (2012) (10) |  |  |  |  |  |  | 4 |
| Clemens et al (2017) (13); 68 | 4 |  |  |  |  |  |  |
| De Vries et al (2012) (14) |  |  |  | 12/69 (17) |  |  | 3 |
| Ditters et al (2023) (16), 100 |  |  |  | 32/121 (26.4)  (Home and Hospital) |  |  |  |
| Hahn et al (2018) (20), 26 |  |  |  |  |  |  |  |
| Harlaar et al (2019) (23), 30 |  |  |  |  | 2 |  | 2 |
| Kuperus et al (2017) (26), 88 |  |  |  | 19/88 (26) |  |  |  |
| Regnery et al (2012) (30); 38 |  |  |  |  | 1 |  | 2 |
| Strothotte et al (2009) (31); 44 |  |  |  |  |  |  | 0 |
| Van der Ploeg (2016) (34); 16 |  | 6/16 (35.5) |  | 4/16 (25) | 0 | 1 |  |
| Van Kooten et al (2020) (36); 111 | 14 (10 were pompe related) |  |  |  |  |  | 10 |

TEAE treatment emergent adverse event; IAR infusion-associated reactions; SAE serious adverse event

**Table 7 Adverse events reported in other prospective ERT studies**

| **Authors** | **Subgroup** | **Adverse events** | | | | | |
| --- | --- | --- | --- | --- | --- | --- | --- |
|  |  | TEAE  n/N (%) | TEAE related to treatment n/N (%) | IAR  n/N (%) | Withdrawal due to TEAE n/N (%) | SAEs  n/N (%) | Discontinuation  n |
| Avalglucosidase alfa studies | | | | | | | |
| Dimachkie et al (2022) (37), 24 | Total | 24 (100) | 18 (75) | 6 (25) | 1 (4) | 9 (38) | 3 |
|  | Naïve | 10 (100) | 8 (80) | 3 (30) | 1 (10) | 5 (50) |  |
|  | Experienced | 14 (100) | 10 (71) | 3 (21) | 0 (0) | 4 (29) |  |
| Cipaglucosidase + Miglustat studies | | | | | | | |
| Byrne et al (2023) (46) | Experienced, 23 | 23 (100) | 16 (70) | 10 (43) | 2 (9) | 8 (35) | 3 |
|  | Naïve, 6 | 6 (100) | 4 (67) | 3 (50) | 0 (0) | 4 (67) |  |

TEAE treatment emergent adverse event; IAR infusion-associated reactions; SAE serious adverse event

**References**

1. Berger KI, Chien Y-H, Dubrovsky A, Kishnani PS, Llerena JC, Neilan E, et al. Changes in forced vital capacity over ≤ 13 years among patients with late-onset Pompe disease treated with alglucosidase alfa: new modeling of real-world data from the Pompe Registry. J Neurol. 2024.

2. Stockton DW, Kishnani P, van der Ploeg A, Llerena J, Jr., Boentert M, Roberts M, et al. Respiratory function during enzyme replacement therapy in late-onset Pompe disease: longitudinal course, prognostic factors, and the impact of time from diagnosis to treatment start. J Neurol. 2020 Oct;267(10):3038-53.

3. Marcondes B, Wilker E, Laredo F, Llerena J, Falavigna M. PRO6 Characteristics of Late Onset Pompe Disease Patients in Latin America - a Patient Registry Analysis. Value Health. [Conference Abstract]. 2020 December;23(Supplement 2):S690-S1.

4. Semplicini C, De Antonio M, Taouagh N, Behin A, Bouhour F, Echaniz-Laguna A, et al. Long-term benefit of enzyme replacement therapy with alglucosidase alfa in adults with Pompe disease: Prospective analysis from the French Pompe Registry. J Inherit Metab Dis. [Multicenter Study Observational Study Research Support, Non-U.S. Gov't]. 2020 11;43(6):1219-31.

5. Lefeuvre C, De Antonio M, Bouhour F, Tard C, Salort-Campana E, Lagrange E, et al. Characteristics of Patients With Late-Onset Pompe Disease in France: Insights From the French Pompe Registry in 2022. Neurology. [Research Support, Non-U.S. Gov't]. 2023 08 29;101(9):e966-e77.

6. Tard C, Bouhour F, Michaud M, Beltran S, Fournier M, Demurger F, et al. Real-life effectiveness 1 year after switching to avalglucosidase alfa in late-onset Pompe disease patients worsening on alglucosidase alfa therapy: A French cohort study. Eur J Neurol. 2024:e16292.

7. Martinez-Marin RJ, Reyes-Leiva D, Nascimento A, Muelas N, Dominguez-Gonzalez C, Paradas C, et al. Description of clinical and genetic features of 122 patients included in the Spanish Pompe registry. Neuromuscul Disord. 2024;34:1-8.

8. Anderson LJ, Henley W, Wyatt KM, Nikolaou V, Waldek S, Hughes DA, et al. Effectiveness of enzyme replacement therapy in adults with late-onset Pompe disease: results from the NCS-LSD cohort study. J Inherit Metab Dis. [Multicenter Study Observational Study Research Support, Non-U.S. Gov't]. 2014 Nov;37(6):945-52.

9. Angelini C, Semplicini C, Ravaglia S, Bembi B, Servidei S, Pegoraro E, et al. Observational clinical study in juvenile-adult glycogenosis type 2 patients undergoing enzyme replacement therapy for up to 4 years. J Neurol. [Multicenter Study]. 2012 May;259(5):952-8.

10. Angelini C, Semplicini C, Ravaglia S, Moggio M, Comi GP, Musumeci O, et al. New motor outcome function measures in evaluation of late-onset Pompe disease before and after enzyme replacement therapy. Muscle & Nerve. [Research Support, Non-U.S. Gov't]. 2012 Jun;45(6):831-4.

11. Bembi B, Pisa FE, Confalonieri M, Ciana G, Fiumara A, Parini R, et al. Long-term observational, non-randomized study of enzyme replacement therapy in late-onset glycogenosis type II. J Inherit Metab Dis. [Clinical Trial Multicenter Study Research Support, Non-U.S. Gov't]. 2010 Dec;33(6):727-35.

12. Claeys KG, D'Hondt A, Fache L, Peers K, Depuydt CE. Six-Minute Walk Distance Is a Useful Outcome Measure to Detect Motor Decline in Treated Late-Onset Pompe Disease Patients. Cells. 2022 01 20;11(3):20.

13. Clemens P, Laforet P, Kacena K, Sanson BJ, Hopkin R, Van DPA. Long-term efficacy of alglucosidase alfa in late-onset pompe disease. Neurology. [Journal article; Conference proceeding]. 2017;88(16).

14. de Vries JM, van der Beek NA, Hop WC, Karstens FP, Wokke JH, de Visser M, et al. Effect of enzyme therapy and prognostic factors in 69 adults with Pompe disease: an open-label single-center study. Orphanet Journal Of Rare Diseases. [Research Support, Non-U.S. Gov't]. 2012 Sep 26;7:73.

15. Ditters IAM, van Kooten HA, van der Beek N, Hardon JF, Ismailova G, Brusse E, et al. Home-Based Infusion of Alglucosidase Alfa Can Safely be Implemented in Adults with Late-Onset Pompe Disease: Lessons Learned from 18,380 Infusions. Biodrugs. 2023 Sep;37(5):685-98.

16. Ditters IAM, van der Beek N, Brusse E, van der Ploeg AT, van den Hout JMP, Huidekoper HH. Home-based enzyme replacement therapy in children and adults with Pompe disease; a prospective study. Orphanet Journal Of Rare Diseases. [Research Support, Non-U.S. Gov't]. 2023 05 08;18(1):108.

17. Gungor D, Kruijshaar ME, Plug I, D'Agostino RB, Hagemans ML, van Doorn PA, et al. Impact of enzyme replacement therapy on survival in adults with Pompe disease: results from a prospective international observational study. Orphanet Journal Of Rare Diseases. [Research Support, Non-U.S. Gov't]. 2013 Mar 27;8:49.

18. Gungor D, Kruijshaar ME, Plug I, Rizopoulos D, Kanters TA, Wens SC, et al. Quality of life and participation in daily life of adults with Pompe disease receiving enzyme replacement therapy: 10 years of international follow-up. J Inherit Metab Dis. [Observational Study Research Support, Non-U.S. Gov't]. 2016 Mar;39(2):253-60.

19. Gungor D, Kruijshaar ME, Plug I, Rizopoulos D, Kanters TA, Wens SC, et al. Quality of Life and Participation in the Daily Life (Activities) of Adults with Pompe Disease Receiving Enzyme Replacement Therapy: 10 Years of International Follow-Up. Journal of neuromuscular diseases. 2015;2(s1):S63.

20. Hahn SH, Kronn D, Leslie ND, Pena LDM, Tanpaiboon P, Gambello MJ, et al. Efficacy, safety profile, and immunogenicity of alglucosidase alfa produced at the 4,000-liter scale in US children and adolescents with Pompe disease: ADVANCE, a phase IV, open-label, prospective study. Genetics in Medicine. [Clinical Trial, Phase IV Research Support, Non-U.S. Gov't]. 2018 10;20(10):1284-94.

21. Harlaar L, Hogrel J, Perniconi B, Kruijshaar M, Rizopoulos D, Taouagh N, et al. O.23A 10 year prospective study on the effects of enzyme replacement therapy in adult Pompe patients. Neuromuscul Disord. [Journal article; Conference proceeding]. 2019;29:S122.

22. Harlaar L, Hogrel JY, Perniconi B, Kruijshaar M, Rizopoulos D, Taouagh N, et al. Variable effects of enzyme replacement therapy in adults with Pompe disease: a 10 year prospective study. Eur J Neurol. [Journal article; Conference proceeding]. 2019;26:40.

23. Harlaar L, Hogrel JY, Perniconi B, Kruijshaar ME, Rizopoulos D, Taouagh N, et al. Large variation in effects during 10 years of enzyme therapy in adults with Pompe disease. Neurology. [Research Support, Non-U.S. Gov't]. 2019 11 05;93(19):e1756-e67.

24. Harlaar L, Hogrel JY, Perniconi B, Kruijshaar ME, Rizopoulos D, Taouagh N, et al. Variable effects of enzyme replacement therapy in adults with Pompe disease: 10 years' follow-up. J Inherit Metab Dis. [Journal article; Conference proceeding]. 2019;42:242.

25. Hartung R, Chamsi-Bacha F, Beck PDmM, Mengel DmE. Initial Therapy Response of 6 Months of Enzyme Replacement Therapy in 11 Juvenile/Adult M. Pompe Patients. Clin Ther. 2007;29(SUPPL. C):S86-S7.

26. Kuperus E, Kruijshaar ME, Wens SCA, de Vries JM, Favejee MM, van der Meijden JC, et al. Long-term benefit of enzyme replacement therapy in Pompe disease: A 5-year prospective study. Neurology. 2017 Dec 05;89(23):2365-73.

27. Kuperus E, van der Meijden JC, In 't Groen SLM, Kroos MA, Hoogeveen-Westerveld M, Rizopoulos D, et al. The ACE I/D polymorphism does not explain heterogeneity of natural course and response to enzyme replacement therapy in Pompe disease. PLoS One. [Clinical Trial Research Support, Non-U.S. Gov't]. 2018;13(12):e0208854.

28. Ravaglia S, de Giuseppe R, Carlucci A, Jehne S, Crescimanno G, Ahmad L, et al. Bioimpedance Phase Angle as a Prognostic Tool in Late-Onset Pompe Disease: A Single-Centre Prospective Study With a 15-year Follow-Up. Frontiers in Cell & Developmental Biology. 2022;10:793566.

29. Ravaglia S, De Filippi P, Pichiecchio A, Ponzio M, Saeidi Garaghani K, Poloni GU, et al. Can genes influencing muscle function affect the therapeutic response to enzyme replacement therapy (ERT) in late-onset type II glycogenosis? Molecular Genetics & Metabolism. [Clinical Trial

Research Support, Non-U.S. Gov't]. 2012 Sep;107(1-2):104-10.

30. Regnery C, Kornblum C, Hanisch F, Vielhaber S, Strigl-Pill N, Grunert B, et al. 36 months observational clinical study of 38 adult Pompe disease patients under alglucosidase alfa enzyme replacement therapy. J Inherit Metab Dis. [Clinical Trial Research Support, Non-U.S. Gov't]. 2012 Sep;35(5):837-45.

31. Strothotte S, Strigl-Pill N, Grunert B, Kornblum C, Eger K, Wessig C, et al. Enzyme replacement therapy with alglucosidase alfa in 44 patients with late-onset glycogen storage disease type 2: 12-month results of an observational clinical trial. J Neurol. [Clinical Trial]. 2010 Jan;257(1):91-7.

32. van der Meijden JC, Kruijshaar ME, Rizopoulos D, van Doorn PA, van der Beek N, van der Ploeg AT. Enzyme replacement therapy reduces the risk for wheelchair dependency in adult Pompe patients. Orphanet Journal Of Rare Diseases. [Research Support, Non-U.S. Gov't]. 2018 05 22;13(1):82.

33. van der Meijden JC, Kruijshaar ME, Harlaar L, Rizopoulos D, van der Beek N, van der Ploeg AT. Long-term follow-up of 17 patients with childhood Pompe disease treated with enzyme replacement therapy. J Inherit Metab Dis. [Research Support, Non-U.S. Gov't]. 2018 11;41(6):1205-14.

34. van der Ploeg A, Carlier PG, Carlier RY, Kissel JT, Schoser B, Wenninger S, et al. Prospective exploratory muscle biopsy, imaging, and functional assessment in patients with late-onset Pompe disease treated with alglucosidase alfa: The EMBASSY Study. Molecular Genetics & Metabolism. [Clinical Trial, Phase IV Multicenter Study Research Support, Non-U.S. Gov't]. 2016 09;119(1-2):115-23.

35. Thurberg BL, Carlier P, Kissel JT, Schoser B, Pestronk A, Barohn RJ, et al. A Phase 4 Prospective Study in Patients with Adult Pompe Disease Treated with Alglucosidase Alfa. Journal of neuromuscular diseases. 2015;2(s1):S72-S3.

36. van Kooten HA, Harlaar L, van der Beek N, van Doorn PA, van der Ploeg AT, Brusse E. Discontinuation of enzyme replacement therapy in adults with Pompe disease: Evaluating the European POmpe Consortium stop criteria. Neuromuscul Disord. [Evaluation Study]. 2020 01;30(1):59-66.

37. Dimachkie MM, Barohn RJ, Byrne B, Goker-Alpan O, Kishnani PS, Ladha S, et al. Long-term Safety and Efficacy of Avalglucosidase Alfa in Patients With Late-Onset Pompe Disease. Neurology. 2022 May 26;26:26.

38. Pena LDM, Barohn RJ, Byrne BJ, Desnuelle C, Goker-Alpan O, Ladha S, et al. Safety, tolerability, pharmacokinetics, pharmacodynamics, and exploratory efficacy of the novel enzyme replacement therapy avalglucosidase alfa (neoGAA) in treatment-naive and alglucosidase alfa-treated patients with late-onset Pompe disease: A phase 1, open-label, multicenter, multinational, ascending dose study. Neuromuscul Disord. [Clinical Trial, Phase I Multicenter Study Research Support, Non-U.S. Gov't]. 2019 03;29(3):167-86.

39. Dimachkie M, Barohn R, Byrne B, Goker-Alpan O, Kishnani P, Ladha S, et al. NEO1 and NEO-EXT studies: Long-term safety and exploratory efficacy of repeat avalglucosidase alfa dosing for 5.5 years in late-onset pompe disease patients. Neurology Conference: 72nd Annual Meeting of the American Academy of Neurology, AAN. [Conference Abstract]. 2020;94(15 Supplement).

40. Dimachkie MM. NEO1/NEO-EXT: Safety and exploratory efficacy of repeat avalglucosidase alfa dosing for up to 6 years in participants with late-onset pompe disease. Muscle Nerve. [Conference Abstract]. 2020 October;62(SUPPL 1):S64.

41. Schoser B, Barohn RJ, Byrne BJ, Goker-Alpan O, Kishnani PS, Ladha S, et al. NEO1/NEO-EXT studies: Trends over time in exploratory efficacy of repeat avalglucosidase alfa dosing for up to 5.5 years in late-onset Pompe disease (LOPD) patients. Eur J Neurol. [Conference Abstract]. 2020 May;27(Supplement 1):486.

42. Schoser B, Barohn R, Byrne B, Goker-Alpan O, Kishnani P, Ladha S, et al. AUTOPHAGIC MYOPATHIES / MYOFIBRILLAR MYOPATHIES / DISTAL MYOPATHIES / POMPE DISEASE: P.03 NEO1/NEO-EXT studies: Safety and exploratory efficacy of repeat avalglucosidase alfa dosing after up to 6 years in late-onset Pompe disease (LOPD). Neuromuscul Disord. [Conference Abstract]. 2020 October;30(Supplement 1):S49.

43. Schoser B, Kishnani P, Bratkovic D, Clemens P, Goker-Alpan O, Ming X, et al. FP.17 Long-term follow-up of cipaglucosidase alfa/miglustat in ambulatory patients with Pompe disease: An open-label phase I/II study (ATB200-02). Neuromuscul Disord. [Conference Abstract]. 2022 October;32(Supplement 1):S73.

44. Mozaffar T, Schoser B, Kishnani P, Bratkovic D, Clemens PR, Goker-Alpan O, et al. Long-term Follow-up of Cipaglucosidase Alfa/Miglustat in Ambulatory Patients with Pompe Disease: An Open-label Phase I/II Study (ATB200-02). Neurology Conference: American Academy Of Neurology Annual Meeting, AAN. [Conference Abstract]. 2023;100(17 Supplement 2).

45. Byrne BJ, Schoser B, Kishnani PS, Bratkovic D, Clemens PR, Goker-Alpan O, et al. Long-term safety and efficacy of cipaglucosidase alfa plus miglustat in individuals living with Pompe disease: an open-label phase I/II study (ATB200-02). J Neurol. 2023 Dec 06;06:06.

46. Byrne B, Diaz-Manera J, Goker-Alpan O, Mozaffar T, Wasfi Y, Das SS, et al. Safety of home administration of cipaglucosidase alfa+miglustat in late-onset Pompe disease: results from multiple clinical trials. Neuromuscul Disord. [Conference Abstract]. 2023 October;33(Supplement 1):S148.
